# Mitochondrial Localization of Cx26 Promotes Colorectal Cancer Malignancy and Exposes a Fatal Vulnerability to Drug Targeting

**DOI:** 10.1002/advs.78117

**Published:** 2026-09-30

**Authors:** Jie Wang, Lu Chen, Jia Qin, Xiaoning Wang, Sunlan Ye, Ning An, Mei Bai, Yan Shen, Xuefeng Wu, Qiang Xu

**Affiliations:** ^1^ State Key Laboratory of Pharmaceutical Biotechnology School of Life Sciences Nanjing Drum Tower Hospital Nanjing University Nanjing China; ^2^ School of Basic Medical Sciences Nanjing Medical University Nanjing China; ^3^ Lee Kong Chian School of Medicine Nanyang Technological University Singapore Singapore

**Keywords:** bortezomib, colorectal cancer, connexin 26, endoplasmic reticulum‐mitochondria crosstalk, epithelial‐mesenchymal transition, protein L‐isoaspartyl/D‐aspartyl O‐methyltransferase

## Abstract

The malignant progression of colorectal cancer (CRC) is closely linked to metastasis and therapy resistance, but underlying mechanisms remain unclear. Here, we identify mitochondrial translocation of connexin 26 (Cx26) as a hallmark of highly malignant metastatic CRC and reveal its critical role in promoting ER‐mitochondria crosstalk, calcium signaling, and epithelial‐mesenchymal transition (EMT). Mechanistically, we demonstrate that protein L‐isoaspartyl/D‐aspartyl O‐methyltransferase (PIMT) methylates Cx26 at asparagine 176, enhancing its mitochondrial localization and stability. This modification disrupts Cx26's gap junction function while activating calcium signaling to drive EMT and metastasis. Furthermore, we discovered that bortezomib (BTZ), contrary to its canonical proteasome inhibition mechanism, exhibits exceptional efficacy against highly malignant CRC by targeting the PIMT‐Cx26 axis. BTZ directly binds PIMT, further amplifying Cx26 mitochondrial translocation and hyperactivating Ca^2^^+^‐ROS‐ERK signaling, ultimately inducing lethal autophagy and apoptosis in metastatic CRC cells. Strikingly, low‐malignancy primary CRC cells remain resistant to BTZ, highlighting its selectivity for advanced disease. Collectively, our study uncovers mitochondrial Cx26 as a novel therapeutic vulnerability in metastatic CRC and repurposes BTZ as a precision strategy targeting PIMT.

## Graphical abstract

Mitochondrial localization of Cx26 emerges as a double‐edged sword in colorectal cancer. PIMT‐mediated methylation of Cx26 promotes its mitochondrial localization and stability. Mitochondrial Cx26 then facilitates ER‐mitochondria communication and Ca^2+^ signaling, thereby promoting metastatic progression. Bortezomib exploits this malignant adaptation by further amplifying the PIMT‐Cx26 interaction, triggering excessive Ca^2+^‐ROS‐ERK signaling and ultimately inducing autophagy and apoptosis, thereby converting a metastatic advantage into a therapeutic vulnerability.

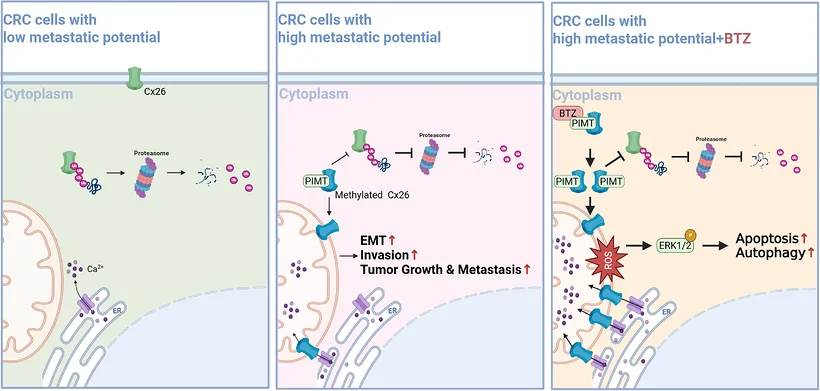

## 1 Introduction

High‐malignancy metastatic colorectal cancer (CRC) is mainly derived from low‐malignancy primary CRC through the blood and lymphatic system. Once metastatic, it is difficult to cure and is more prone to recurrence. 40%–50% of CRC patients present with metastasis at diagnosis or develop recurrence, and most CRC patients with distant metastases have a five‐year survival rate of less than 10% [1]. At present, metastasis and drug resistance remain challenges in CRC treatment [2]. The malignant progression of tumors is often accompanied by changes in some molecular properties. Epithelial‐mesenchymal transition (EMT) is a critical process for epithelial cell‐derived malignancies to gain the ability to invade and migrate, which is often accompanied by the cell junction transition from E‐cadherin to N‐cadherin, so that epithelial cells lose cell connectivity and polarity and transform into mesenchymal cells with infiltrative and migratory capabilities [3, 4, 5]. Tumor resistance is also strongly associated with EMT. Recurrent tumors have significant EMT‐like features, and deletion of E‐cadherin promotes drug resistance in CRC patients [6, 7]. This suggests that CRC needs to alter the expression and function of a number of molecules to achieve malignant progression, including metastasis and drug resistance. Therefore, it is of great significance to identify the key differential molecular events between highly malignant metastatic CRC and less malignant primary CRC, explore the mechanism of CRC malignant progression, and provide new strategies and methods for the clinical treatment of highly malignant metastatic CRC.

Connexin 26 (Cx26), encoded by *GJB2*, is a gap junction protein. Six homologous or heterologous connexins can oligomerize to form hemichannels that connect material exchange between cells or between cells and the extracellular environment, thereby regulating tissue development, homeostasis, and various cellular functions [8, 9]. Cx26 was previously thought to be a tumor suppressor, but many studies now also show that increased Cx26 expression is associated with metastasis and poor prognosis in a variety of human malignancies [10, 11]. Cx26 has been proposed as a prognostic biomarker and therapeutic target for lung adenocarcinoma, and it can also transmit radiation damage signals and regulate the radiosensitivity of cutaneous squamous cell carcinoma by activating the MAPK/NF‐κB signaling pathway [12]. Studies have reported that normal epithelium and adenomatous tissue in colon polyps express Cx26 on the plasma membrane, while cancer tissue mainly displays cytoplasmic Cx26 accumulation [13]. Compared with normal liver tissue, Cx26 in hepatocellular carcinoma cells tends to be located in the cytoplasm [14]. Cx26 was also found to be highly expressed in self‐renewing cancer stem cells (CSCs), and it forms a signaling complex with the pluripotency transcription factor NANOG and focal adhesion kinase (FAK) to promote CSC self‐renewal by stabilizing NANOG and activating FAK, but this signaling complex is not formed in mammary epithelial or luminal breast cancer cells [15]. The accumulation of Cx26 in the cytoplasm may constitute indirect evidence for the lack of functional gap junction channels between cancer cells, thereby promoting local invasion of tumor cells. These studies suggest an important role of Cx26 in tumor metastasis, but its mechanism of action remains to be elucidated.

Protein L‐isoaspartyl/D‐aspartyl O‐methyltransferase (PIMT), encoded by *PCMT1*, is a protein repair enzyme that specifically methylates the isoaspartyl residues produced by the spontaneous deamidation of asparagine, which is the necessary step for the conversion of isoaspartic acid to aspartic acid [16]. Deamidation of asparagine can impair protein function and contribute to cellular aging [17]. Therefore, PIMT is essential for maintaining normal protein conformation and function. Dysregulated PIMT expression has been associated with tumor progression and poor prognosis in several cancers, including bladder cancer and lung adenocarcinoma [18]. However, whether PIMT contributes to CRC progression and, in particular, whether it regulates the abnormal localization and stability of Cx26 in aggressive CRC, remains unclear.

Given that Cx26 is highly expressed in aggressive CRC, we asked whether its expression level could predict sensitivity to clinically relevant drugs, and we therefore queried the GDSC1 and GDSC2 datasets across 23 CRC cell lines. In our study, we found that Cx26 was highly expressed and abundantly localized to mitochondria in highly malignant metastatic CRC cells, and that PIMT‐mediated modification of Cx26 at N176 may contribute to its mitochondrial localization and protein stability. Given the potential role of Cx26 in CRC cell EMT and malignant progression, we hypothesized that Cx26 may represent a therapeutically actionable target in highly aggressive CRC. Despite advances in targeted therapies, metastatic CRC often develops resistance to conventional drugs such as 5‐fluorouracil and oxaliplatin [2]. To identify clinically relevant compounds that may preferentially target this malignant state, we leveraged the Genomics of Drug Sensitivity in Cancer (GDSC1 and GDSC2) databases to systematically evaluate the relationship between GJB2 expression and drug sensitivity across 23 CRC cell lines. Intriguingly, although higher GJB2 expression was generally associated with reduced sensitivity to most tested chemotherapeutic agents, the 26S proteasome inhibitor bortezomib (BTZ) showed an inverse association, with CRC cells exhibiting higher GJB2 expression displaying lower IC_50_ values. In addition, we found that BTZ interacted with PIMT. This association prompted us to investigate whether BTZ preferentially suppresses Cx26‐high CRC cells by interfering with the PIMT‐Cx26 regulatory axis. In this study, we identify mitochondrial Cx26 as a PIMT‐dependent driver of CRC EMT and malignant progression and further reveal a previously unrecognized mechanism underlying the activity of BTZ in Cx26‐high CRC cells. These findings provide a potential rationale for targeting the PIMT‐Cx26 axis as a therapeutic strategy for highly aggressive metastatic CRC.

## 2 Materials and Methods

### 2.1 Chemicals and Reagents

Cell lysis buffer for Western blotting and immunoprecipitation, RIPA lysis buffer, anti‐HSP60 antibody, NAC, and penicillin‐streptomycin solution (100×) were provided by Beyotime Biotechnology (Shanghai, China). Anti‐Cx26 antibody, anti‐Calnexin antibody, and anti‐Flag antibody were purchased from Proteintech Group (CHI, USA). Anti‐N‐cadherin antibody, anti‐Cx26 antibody, and ruthenium red were purchased from Abcam (Cambridge, UK). Antibodies against Vimentin, phospho‐ERK1/2, E‐cadherin, PARP, LC3B, BCL2, and Na, K‐ATPase were purchased from Cell Signaling Technology (Danvers, MA, USA). Antibodies against β‐Tubulin, β‐Actin, HA, His, and GAPDH were purchased from Abmart (Shanghai, China). Anti‐COX4, anti‐calreticulin, anti‐GM130, anti‐EEA1, anti‐Ub, anti‐PIMT, anti‐IP3R‐I/II/III, anti‐VDAC1 and anti‐TOM20 antibodies were purchased from Santa Cruz Biotechnology (CA, USA). Histamine, bortezomib, irinotecan, chloroquine, cycloheximide, MCU‐i4 and PD0325901 were provided by MedChemExpress (NJ, USA). MG132 was obtained from Topscience (Shanghai, China). 5‐Fluorouracil, oxaliplatin, paclitaxel, and etoposide were obtained from Aladdin Bio‐Chem Technology (Shanghai, China).

### 2.2 Animal Experiments

The animal study was reviewed and approved by the Nanjing University Animal Care and Use Committee (IACUC‐D2302115). Six‐ to eight‐week‐old male BALB/c nude mice were purchased from Si Pei Fu Biotechnology Co., Ltd. (Beijing, China). Male C57BL/6 mice aged 6–8 weeks were purchased from Beijing Vital River Laboratory Animal Technology Co., Ltd. (Beijing, China). SW480 (NC and OE‐Cx26), SW620 (shRNA‐NC and shRNA‐Cx26), and B16F10 (shRNA‐NC and shRNA‐Cx26) cells were collected and washed three times with PBS. After cell counting, B16F10 (shRNA‐NC or shRNA‐Cx26) cells (1 × 10^6^ cells in 100 µL PBS), SW480 (NC or OE‐Cx26) and SW620 (shRNA‐NC or shRNA‐Cx26) cells (1 × 10^7^ cells in 100 µL PBS) were subcutaneously inoculated into each mouse (*n* = 5 or 6 mice per group). After inoculation, the mice were observed for 5–7 days to confirm the success of tumor implantation. Then, tumor length and width were measured every other day, and tumor volume (mm^3^) = length (mm) × width [2] (mm^2^) × 0.5 was calculated. According to the tumor volume, the mice were euthanized at an appropriate time, and the materials and subsequent experiments were carried out.

Four‐ to five‐week‐old male BALB/c nude mice and male C57BL/6 mice aged 6–8 weeks were purchased from Beijing Vital River Laboratory Animal Technology Co., Ltd. (Beijing, China). Tail vein injection‐induced experimental metastasis models were established using SW480, SW620, and B16F10 cells. Mice received tail vein injections of SW480‐NC/OE‐Cx26 or SW620‐shRNA‐NC/shRNA‐Cx26 cells (2.5 × 10^6^ cells in 100 µL per mouse, *n* = 6/group), or B16F10‐shRNA‐NC/shRNA‐Cx26 cells (1 × 10^6^ cells in 100 µL per mouse; *n* = 5/group). To evaluate the effect of BTZ on CRC metastasis in vivo, an experimental metastasis model was established by tail vein injection of SW620 cells (2.5 × 10^6^ cells in 100 µL per mouse; *n* = 6 per group). Mice received four intraperitoneal injections of BTZ (1 mg/kg) during the 56‐day experimental period. Body weight and general condition were monitored throughout the experiments. Mice were euthanized at 42, 56, and 21 days after injection for the SW480, SW620, and B16F10 models, respectively, and tissues were collected for subsequent analyses.

### 2.3 Western Blot and Coimmunoprecipitation

Cells or tissues were lysed in lysis buffer containing a protease inhibitor cocktail for 30 min on ice, and supernatants were collected after centrifugation of cell lysates at 12 000 × g for 10 min. The remaining steps are consistent with previous reports [19]. The protocols for coimmunoprecipitation have been reported previously [20].

### 2.4 Real‐Time PCR

The protocols have been reported previously [19]. The sequences of primers used in our study are as follows: GJB2 forward, 5’‐TCG CAT TAT GAT CCT CGT TGT G‐3’; GJB2 reverse, 5’‐GGG GAA GTA GTG ATC GTA GCA C‐3’; PCMT1 forward, 5’‐AGG AAG GAC GAT CCA ACA CTT‐3’; PCMT1 reverse, 5’‐GGC TTC TTC AGC ATA TCC CAT T‐3’; CDH1 forward, 5’‐CGA GAG CTA CAC GTT CAC GG‐3’; CDH1 reverse, 5’‐GGG TGT CGA GGG AAA AAT AGG‐3’; VIM forward, 5’‐GAC GCC ATC AAC ACC GAG TT‐3’; VIM reverse, 5’‐CTT TGT CGT TGG TTA GCT GGT‐3’; BCL2L1 forward, 5’‐GAC TGA ATC GGA GAT GGA GAC C‐3’; BCL2L1 reverse, 5’‐GCA GTT CAA ACT CGT CGC CT‐3’; MDR1 forward, 5’‐GGG ATG GTC AGT GTT GAT GGA‐3’; MDR1 reverse, 5’‐GCT ATC GTG GTG GCA AAC AAT A‐3’; p62 forward, 5’‐GAC TAC GAC TTG TGT AGC GTC‐3’; p62 reverse, 5’‐AGT GTC CGT GTT TCA CCT TCC‐3’; GAPDH forward, 5’‐CTC TCT GCT CCT CCT GTT CGA C‐3’; GAPDH reverse, 5’‐TGA GCG ATG TGG CTC GGC T‐3’.

### 2.5 Cell Culture

The SW480 cell line, SW620 cell line, B16F1 cell line, B16F10 cell line, and HEK293T cell line were obtained from the National Collection of Authenticated Cell Cultures (Shanghai, China). SW480, SW620, B16F1, and B16F10 cells were cultured in RPMI 1640 medium supplemented with penicillin‐streptomycin solution (1×) and 10% fetal bovine serum (FBS) under an atmosphere of 5% (*v*/*v*) CO_2_ at 37°C. HEK293T cells were cultured in DMEM containing penicillin‐streptomycin solution (1×) and 10% FBS under an atmosphere of 5% (*v*/*v*) CO_2_ at 37°C.

To mimic the induction and progression of epithelial‐mesenchymal transition (EMT) in tumor cells, SW480 cells were stimulated with 10 ng/ml recombinant human TGF‐β (PeproTech, Rocky Hill, USA) for 24, 48, or 72 h, followed by subsequent analyses.

### 2.6 Plasmids and Lentivirus Transfection

Empty Vector, Cx26‐HA, Cx26‐Flag, Cx26 Δ1‐40‐HA, Cx26 N176A‐HA, PIMT‐Flag, PIMT G88A‐Flag, and Ub‐HA plasmids were constructed by Sangon Biotech Co., Ltd. (Shanghai, China), and the vector was pcDNA3.1. Ub‐His and pCMV‐CEPIA4‐mit plasmids were purchased from Miaoling Biotechnology Co., Ltd. (Wuhan, China). The negative control shRNA‐NC lentivirus, human shRNA‐Cx26 lentivirus, and mouse shRNA‐Cx26 lentivirus were constructed by Genechem Co., Ltd. (Shanghai, China). The non‐targeting control shRNA (shRNA‐NC) sequence was 5’‐TTC TCC GAA CGT GTC ACG T‐3’, the human shRNA‐Cx26 target sequence was 5’‐ACG TGT GCT ACG ATC ACT A‐3’, the mouse shRNA‐Cx26 target sequence was 5’‐GCT ATT TGT TCG TTA GGT ATT‐3’, and the vector was hU6‐MCS‐CMV‐Puromycin. PLenti‐EF1a‐EGFP‐P2A‐Puro‐CMV‐GJB2‐3Flag lentivirus and a negative control virus were constructed by Obio Technology Co., Ltd. (Shanghai, China). ShRNA‐PIMT was constructed by Genechem Co., Ltd. (Shanghai, China); the target sequence was 5’‐GCG CTA GAA CTT CTA TTT GAT‐3’, and the vector was hU6‐MCS‐CMV‐puromycin. SW480 and SW620 cells were transfected with plasmids using Lipofectamine 3000 Transfection Reagent (Thermo Fisher Scientific, MA, USA). HEK293T cells were transfected with plasmids using Exfect Transfection Reagent (Vazyme, Nanjing, China).

### 2.7 Preparation of the Subcellular Fractions

Cytoplasmic and membrane fractions were prepared using the Mem‐PER Plus Membrane Protein Extraction Kit (Thermo Fisher Scientific, MA, USA) or Minute Plasma Membrane Protein Isolation and Cell Fractionation Kit (Invent, MN, USA) according to the manufacturer's instructions. Mitochondrial fractions were extracted according to the instructions of the Cell Mitochondria Isolation Kit (Beyotime, Shanghai, China) or the method previously reported [21]. Endoplasmic reticulum (ER) fractions were prepared by using a Minute ER Enrichment Kit for Tissues and Cultured Cells (Invent, MN, USA) according to the manufacturer's instructions. Golgi fractions were prepared by using a Minute Golgi Apparatus Enrichment Kit (Invent, MN, USA) according to the manufacturer's instructions. Endosomal fractions were prepared by using a Minute Endosome Isolation Kit (Invent, MN, USA) according to the manufacturer's instructions.

### 2.8 Immunohistochemistry

Tissue sections were deparaffinized, rehydrated, antigen retrieved, blocked, and then incubated overnight at 4°C with antibodies against PIMT. The remaining steps are consistent with previous reports [19].

### 2.9 Immunofluorescence Assay

Cells stained with or without MitoTracker Red (Beyotime, Shanghai, China) were fixed, permeabilized, blocked, and then incubated with anti‐Cx26 and/or anti‐PIMT antibodies overnight at 4°C. After washing with PBS, the cells were exposed to Alexa Fluor 488‐conjugated anti‐rabbit IgG (Thermo Fisher Scientific, MA, USA) and/or Alexa Fluor 594‐conjugated anti‐mouse IgG (Thermo Fisher Scientific, MA, USA), and finally, the nuclei were stained with DAPI.

Multiplex immunofluorescence (mIF) staining was performed on the colorectal cancer (CRC) tissue microarray using a Three‐Target, Four‐Color Multiplex Fluorescence Staining Kit (AiFang Biological, Hunan, China) according to the manufacturer's instructions. Briefly, tissue sections were deparaffinized, rehydrated, subjected to antigen retrieval, and blocked, followed by sequential incubation with primary antibodies against Cx26 and TOM20. Signal amplification was performed using a TSA‐based multiplex fluorescence staining system, with antibody stripping and reblocking between successive staining rounds. Finally, nuclei were counterstained with DAPI, and the sections were mounted for fluorescence imaging. Fluorescence images were analyzed using Fiji. The mitochondrial co‐localization of Cx26 was quantified using Manders’ M1 coefficient, defined as the fraction of the Cx26 fluorescence signal overlapping with TOM20‐positive mitochondria.

### 2.10 Cellular Thermal Shift Assay

After treatment of SW620 cells with DMSO or BTZ (100 nM) for 2 h, cell lysates were collected, divided into 10 aliquots, heated at the indicated temperatures, and then subjected to western blotting.

### 2.11 Surface Plasmon Resonance

PCMT1 Human Recombinant Protein (Origene, MD, USA) was immobilized on an activated Series S Sensor Chip CM5 chip, and a gradient concentration of BTZ solution was prepared. The Biacore T200 molecular interaction analysis system (GE Healthcare, Uppsala, Sweden) was used to measure the change in response value when the gradient concentration of BTZ solution flowed through the chip, and the kinetic constant of the interaction between small molecules and proteins was obtained.

### 2.12 Differential Scanning Fluorimetry

One microliter of human PCMT1 recombinant protein (75 ng/µL, purity >80%), 1 µL of SYPRO Orange (10×), negative control DMSO or BTZ (final concentrations of 0.01, 0.1, 1 µM) or positive control SAH (final concentration of 1 µM) was added to the wells of real‐time PCR plates, and the total volume was made up to 10 µL with citrate buffer. Then, the real‐time PCR plate was put into a Bio‐Rad CFX96 Touch real‐time PCR detection system (Bio‐Rad, Hercules, CA, USA). The reaction conditions were as follows: 30–90°C continuous temperature rise (1°C/min). After the reaction, the protein denaturation temperature of the BTZ group and SAH group was compared with that of the DMSO group to obtain the delta Tm.

### 2.13 Flow Cytometry

Cells were collected and stained according to the instructions of the Annexin V‐FITC Apoptosis Detection Kit (Beyotime, Shanghai, China), Fluo‐4 AM (Beyotime, Shanghai, China), or Reactive Oxygen Species Assay Kit (Beyotime, Shanghai, China) and then detected by a BD FACSCalibur flow cytometer.

### 2.14 Cell Viability

Cell viability was determined using MTT (Beyotime, Shanghai, China).

### 2.15 Subcellular Calcium Monitoring

The protocols have been reported previously [22]. Cells were transfected with the pCMV‐CEPIA4‐mit plasmid for 24–48 h and detected every 30 s using a fluorescence microplate reader (Ex: 488 nm, Em: 507 nm). The cells were then stimulated with 20 µM histamine and detected every 30 s using a fluorescence microplate reader.

### 2.16 Electron Microscopy

After the samples were fixed with prefixed electron microscopy fixative (Servicebio, Wuhan, China), they were sent to Wuhan Servicebio Co., Ltd. (Wuhan, China) to complete the electron microscopy scan.

### 2.17 Transwell Invasion Assay

Twenty‐four‐well Transwell inserts with an 8 µm pore size (Beyotime Biotechnology, Shanghai, China) were precoated with 50 µL of Matrigel Basement Membrane Matrix (REF 356237, Corning, Bedford, MA, USA), diluted in serum‐free medium to a final concentration of 1 mg/ml, and incubated at 37°C for 1 h to allow polymerization. SW480‐NC and SW480‐OE‐Cx26 cells in the logarithmic growth phase were harvested, resuspended in serum‐free medium, and seeded into the upper chambers at a density of 1 × 10^5^ cells per well. The lower chambers were filled with 600 µL of complete medium containing 20% fetal bovine serum (FBS) as a chemoattractant. After incubation for 24 h at 37°C in a humidified atmosphere containing 5% CO_2_, non‐invading cells remaining on the upper surface of the membrane were gently removed using a cotton swab. Cells that had invaded through the membrane to its lower surface were fixed with 4% paraformaldehyde for 30 min, stained with 0.1% crystal violet for 20 min, thoroughly washed with PBS, and photographed under a light microscope (Olympus, Tokyo, Japan). The number of invading cells was quantified by counting cells in five randomly selected fields per insert.

### 2.18 Gap Junction Function Assay

CM‐Dil (red fluorescent dye; Yeasen Biotechnology Co., Ltd., Shanghai, China) and Calcein‐AM (green fluorescent dye; Yeasen Biotechnology Co., Ltd., Shanghai, China) stock solutions were prepared in DMSO at concentrations of 5 mM and 2 mM, respectively. A total of 5 × 10^5^ cells were seeded in confocal dishes and designated as recipient cells. Meanwhile, 1 × 10^5^ cells were seeded in 24‐well plates and designated as donor cells. After 24 h of culture, the culture medium of the donor cells was removed, and the cells were washed three times with PBS. The cells were then incubated with a labeling solution consisting of 0.5 mL of culture medium, 1 µL of 5 mM CM‐Dil, and 1 µL of 2 mM Calcein‐AM for 30 min in a cell culture incubator. The labeling solution was subsequently removed, and the cells were washed three times with PBS. The donor cells were then detached by trypsinization, counted, and 500 donor cells were added to each confocal dish containing the recipient cells. The donor and recipient cells were co‐cultured for 3 h and subsequently examined using fluorescence microscopy.

Images from all experimental groups were acquired using identical fluorescence acquisition settings. For quantitative analysis, four to five randomly selected, non‐overlapping fields were analyzed per dish. CM‐Dil‐positive cells were identified as donor cells, whereas recipient cells exhibiting Calcein fluorescence above the background threshold were considered dye‐positive receiver cells. The fluorescence threshold was established based on background fluorescence and was kept constant for all images within the same experiment. Only Calcein‐positive recipient cells in direct contact with a CM‐Dil‐positive donor cell were included. GJIC was quantified as the mean number of Calcein‐positive recipient cells per CM‐Dil‐positive donor cell. Quantification was performed using images coded to conceal the experimental group from the investigator.

### 2.19 PIMT Enzymatic Activity Assay

According to the manufacturer's instructions for the methyltransferase activity assay kit (BioVision, Milpitas, CA, USA), bovine γ‐globulin was used as the substrate for PIMT at a final concentration of 1 mg/mL. Each reaction contained 1 µg of recombinant PIMT protein, DMSO or BTZ at different concentrations (final concentrations of 0.1 and 1 µM), and 50 µL of Sample Reaction Buffer, consisting of 37 µL of MT Assay Buffer, 2 µL of Enzyme Mix I, 2 µL of Enzyme Mix II, 6 µL of Enzyme Mix III, 1 µL of SAM Cofactor, and 2 µL of Red Probe. The total reaction volume was 100 µL, with the remaining volume supplemented with the Assay Buffer provided in the kit. The reactions were incubated at 37°C for 45 min. A standard curve was generated using the SAH standard, and the OD values obtained from the experimental samples were converted to the corresponding SAH amounts according to the standard curve. Methyltransferase activity was calculated using the following formula: Specific activity = (Sample Reaction Slope/PIMT amount) (µU/µg).

### 2.20 Clinical Samples

The human colon cancer tissue microarray (HCol‐Ade080CD‐01) was provided by Shanghai Outdo Biotech Co., Ltd. This study was approved by the Ethics Committee of Shanghai Outdo Biotech Co., Ltd. (Ethics Approval No. SHYJS‐CP‐1404007).

### 2.21 Statistical Analysis

Data are presented as the mean ± SEM. Statistical analyses were performed using GraphPad Prism 8 (San Diego, CA, USA). Comparisons between two independent groups were performed using an unpaired Student's *t*‐test. Comparisons among multiple groups were performed using one‐way ANOVA or Welch's one‐way ANOVA, as appropriate, followed by Dunnett's or Dunnett's T3 multiple comparisons test, as appropriate. Experiments involving two independent factors were analyzed using two‐way ANOVA, followed by Dunnett's or Šídák's multiple comparisons test, as appropriate. Longitudinal tumor growth data were analyzed using two‐way repeated‐measures ANOVA with the Greenhouse‐Geisser correction. *P* < 0.05 was considered significant.

## 3 Results

### 3.1 Cx26 Translocates to Mitochondria, Accompanied by the Malignant Progression of Colorectal Cancer

To explore the differences in the expression levels of Cx26 in tumor tissues and normal tissues, we first performed bioinformatics analysis. The results showed that the expression level of Cx26 was significantly higher in tumor tissues than in adjacent nontumor tissues of CRC patients (Figure 1A). In addition, according to the analysis results of the TCGA database, we found that the expression of Cx26 increased with increasing CRC stage (Figure 1B). Analysis of two GEO databases (GEO database ID: 17536, 17538) showed that the overall survival of CRC patients with high expression of Cx26 was significantly lower than that of CRC patients with low expression of Cx26 (Figure S1). These results suggest that Cx26 is closely associated with the malignant progression of human CRC and is a poor prognostic factor in CRC patients.

**FIGURE 1:**
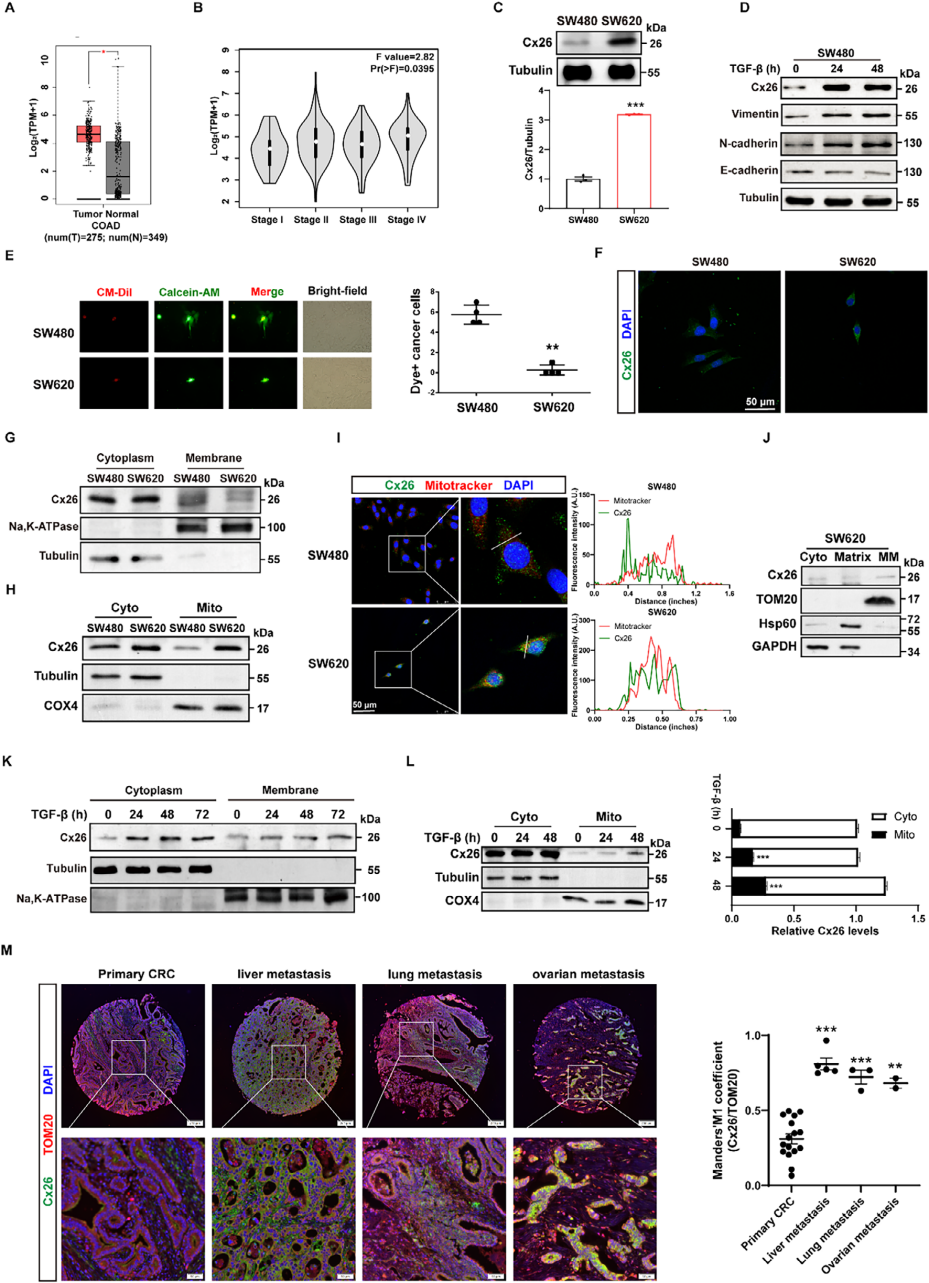
Cx26 translocates to mitochondria, accompanied by the malignant progression of colorectal cancer. (A) The expression levels of Cx26 in CRC and normal colorectal tissue. (B) The expression levels of Cx26 in different CRC stages. (C) The expression levels of Cx26 and Tubulin in primary SW480 or metastatic SW620 cells were analyzed by western blot and statistical analysis, and Tubulin was used as a loading control. ^***^ *p* < 0.001 *vs*. SW480; by unpaired student's *t*‐test (mean ± SEM). (D) The expression of Cx26, Vimentin, N‐cadherin, E‐cadherin, and Tubulin was detected by western blot when stimulated with TGF‐β (10 ng/ml) for 24 or 48 h in SW480 cells, and Tubulin was used as a loading control. (E) The GJIC function of SW480 and SW620 cells was tested, and the number of receiver cells around each donor cell was calculated. ^**^ *p* < 0.01 *vs*. SW480; by unpaired student's *t*‐test (mean ± SEM). (F) Immunofluorescence staining of Cx26 (green) in SW480 and SW620 cells. Nuclei were stained with DAPI (blue). Scale bar: 50 µm. (G) Western blot of Cx26, Na, K‐ATPase, and Tubulin protein levels in the cytoplasm and membrane of SW480 and SW620 cells. Na, K‐ATPase, and Tubulin were measured as loading control of membrane and cytoplasm, respectively. (H) Western blot of Cx26, COX4, and Tubulin protein levels in cytoplasmic and mitochondria‐enriched fractions of SW480 and SW620 cells. Tubulin and COX4 were used as markers and loading controls for the cytoplasmic and mitochondria‐enriched fractions, respectively. (I) Immunofluorescence staining of Cx26 (green) in SW480 and SW620 cells. Mitochondria were stained with MitoTracker Red (red). Nuclei were stained with DAPI (blue). Scale bar: 50 µm. (J) Immunoblot of Cx26, TOM20, Hsp60 and GAPDH in the cytoplasm and submitochondrial fractions of SW620 cells. TOM20, Hsp60 and GAPDH were measured as loading controls of the mitochondrial outer membrane (MM), mitochondrial matrix and cytoplasm, respectively. (K) SW480 cells were treated with TGF‐β (10 ng/ml) for 0, 24, 48, and 72 h; proteins from the cytoplasm and plasma membrane were isolated, and the expression of Cx26 was measured by western blot. Tubulin and Na, K‐ATPase were detected as loading control of cytoplasm and plasma membrane, respectively. (L) Immunoblot and statistical analysis of Cx26, Tubulin, and COX4 protein levels in cytoplasmic and mitochondria‐enriched fractions of SW480 cells incubated with TGF‐β (10 ng/ml) for 0, 24, or 48 h. Tubulin and COX4 were used as markers and loading controls for the cytoplasmic and mitochondria‐enriched fractions, respectively. ^***^ *p* < 0.001 *vs*. 0 h; by One‐way ANOVA followed by Dunnett's multiple comparisons test (mean ± SEM). (M) Multiplex immunofluorescence staining and statistical analysis of Cx26 (green) and TOM20 (red) in a colon cancer tissue microarray. Nuclei were stained with DAPI (blue). ^**^ *p* < 0.01, ^***^ *p* < 0.001 *vs*. primary CRC; by One‐way ANOVA followed by Dunnett's multiple comparisons test (mean ± SEM).

Here, to raise comparability, SW480 and SW620 cell lines were used, which were derived from the same Caucasian male with CRC. SW480 was the primary colon adenocarcinoma, whereas SW620 was isolated from a lymph node metastasis of the same patient after a year. First, we compared the malignancy of SW480 and SW620 cell lines from several aspects, such as proliferative potential, metastatic potential, and stemness, and we found that SW620 was more malignant than SW480 (Figure S2). At the same time, Western blot results showed that SW620 cells expressed more Cx26 than SW480 cells (Figure 1C). When we treated SW480 cells with TGF‐β to induce EMT, the protein level of Cx26 increased (Figure 1D). Cx26 is a member of the gap junction protein family, which can form gap junction channels through which small molecules can pass. Therefore, we next detected the gap junction function of SW480 and SW620 cells by fluorescence tracing. We found that the gap junction function of SW480 cells was stronger than that of SW620 cells, which contradicts the higher expression of Cx26 in SW620 cells than in SW480 cells (Figure 1E). When we used flow cytometry to detect the expression of Cx26 on the cell surface, both NCM460, a human normal colon cell line, and SW480 expressed Cx26 on the cell surface, but SW620 rarely did on the cell surface (Figure S3A). Western blot and immunofluorescence staining showed that Cx26 was more localized in the cytoplasm in SW620 cells than in SW480 cells (Figure 1F,G). These results suggest that Cx26 is mainly located in the cytoplasm and does not function as a gap junction protein in highly malignant SW620 cells.

Since this cytoplasmic pool may be associated with a specific organelle, and given the emerging role of ER‐mitochondria contact sites in calcium signaling and tumor progression, we next explored the intracellular localization of Cx26 using the Protein Subcellular Localization Prediction Tool II (PSORT II) website. The results showed that in addition to the cell membrane, the probability of Cx26 localization in the ER was 55.6%, the probability of Cx26 localization in mitochondria was 33.3%, and the probability of Cx26 localization in secretory vesicles was 11.1% (data not shown). We then isolated multiple organelle proteins from SW480 and SW620 cells, including mitochondrial, endoplasmic reticulum (ER), Golgi, and endosomal proteins. Western blot results showed that compared with SW480 cells, Cx26 abundance was increased in the mitochondrial‐enriched fraction but decreased in the ER‐enriched fraction, with no significant difference in the Golgi‐ and endosome‐enriched fractions (Figure 1H and Figure S3B). Immunofluorescence analysis further demonstrated increased colocalization of Cx26 with mitochondria in SW620 cells (Figure 1I). Cx26 was detectable in both mitochondrial matrix‐ and membrane‐enriched fractions (Figure 1J and Figure S3C). Furthermore, TGF‐β treatment increased Cx26 abundance in the mitochondrial‐enriched fraction of SW480 cells (Figure 1K,L). We also performed colocalization analysis of Cx26 and TOM20 in a human colorectal cancer tissue microarray. In primary colorectal cancer cells, a substantial proportion of Cx26 was localized to the cell membrane, with relatively low colocalization with TOM20. In contrast, Cx26‐TOM20 colocalization was significantly higher in colorectal cancer cells from liver, lung, and ovarian metastatic lesions than in those from primary tumors (Figure 1M).

To determine whether this intriguing phenomenon was not limited to the SW480/SW620 cell pair, we further examined the B16F1 and B16F10 mouse melanoma cell lines, which were derived from the same parental line and share a highly similar genetic background. B16F1 is a relatively poorly metastatic subline, whereas B16F10 is a highly metastatic subline with a strong propensity for pulmonary metastasis. Compared with B16F1 cells, B16F10 cells exhibited lower E‐cadherin expression and higher N‐cadherin and Vimentin expression, indicating a more pronounced EMT phenotype (Figure S3D). Importantly, compared with B16F1 cells, B16F10 cells exhibited lower Cx26 abundance at the cell membrane but higher Cx26 abundance in the mitochondrial‐enriched fraction (Figure S3E,F). Taken together, these findings support the notion that increased mitochondrial translocation of Cx26 is a characteristic associated with a more aggressive and metastatic tumor phenotype.

### 3.2 PIMT Promotes Mitochondrial Translocation of Cx26 in Highly Malignant CRC Cells

The localization of proteins requires guidance of localization tags for their processing and transport. The most common tag is the signal peptide, which is usually located at the N‐terminus of the protein. To explore the mechanism of mitochondrial localization of Cx26, we used SignalP‐5.0 to predict the signal peptide of Cx26, and the results showed that amino acids 1–40 of Cx26 may be its signal peptide (Figure S4A). We transfected the full‐length Cx26‐HA plasmid and Cx26 ∆1‐40‐HA plasmid into HEK293T cells to separate cell membrane proteins. However, the truncation of amino acids 1–40 of Cx26 had no effect on the localization of Cx26 (Figure S4B). We then speculate that the mitochondrial localization of Cx26 may be affected by its interacting proteins. We performed a co‐immunoprecipitation (IP) assay coupled with liquid chromatography‐mass spectrometry (LC‐MS) to identify Cx26‐interacting proteins. PIMT is a candidate among the 118 differentially interacting proteins. Co‐IP analysis with HEK293T cells confirmed the interaction of Cx26 with PIMT (Figure 2A). The results of GEPIA database analysis showed that *PCMT1* and *GJB2* expression were positively correlated (Figure 2B). Furthermore, increased expression of PIMT in SW620 cells was observed compared with that in SW480 cells (Figure 2C), which was markedly associated with malignant progression and poor prognosis in CRC patients (Figure S5). Immunohistochemical results of the human CRC tissue chip also verified that the protein level of PIMT was positively correlated with the malignant progression of CRC (Figure S6). Moreover, the interaction between Cx26 and PIMT in SW480 cells was different from that in SW620 cells. It should be noted that the interaction between PIMT and Cx26 was much stronger in SW620 cells than in SW480 cells, and the interaction in SW480 cells could be enhanced after TGF‐β stimulation (Figure 2D–F). Consistent with these findings, we also observed a stronger interaction between PIMT and Cx26 in B16F10 cells than in B16F1 cells (Figure S7A).

**FIGURE 2:**
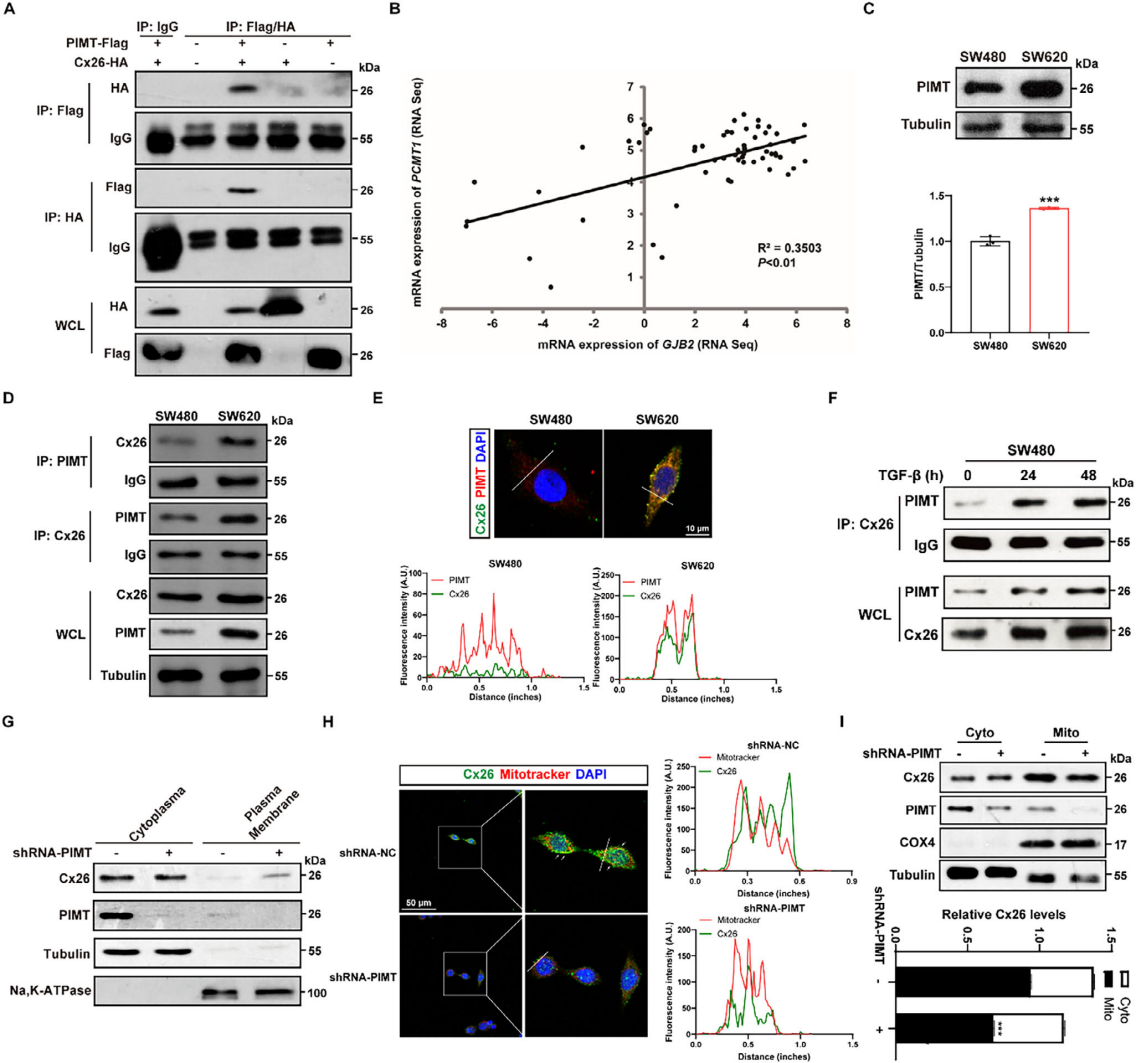
PIMT promotes mitochondrial translocation of Cx26 in highly malignant CRC cells. (A) HEK293T cells were transfected with Cx26‐HA and PIMT‐Flag, and then immunoprecipitated with IgG as a control or anti‐Flag antibody or anti‐HA antibody, followed by western blot for the indicated proteins. (B) Analyze the correlation between the mRNA levels of PIMT and Cx26 in colorectal cancer cell lines through the TCGA database. (C) Immunoblot of PIMT and Tubulin in SW480 and SW620 cells. Tubulin was used as a loading control. ^***^ *p* < 0.001 *vs*. SW480; by unpaired student's *t*‐test (mean ± SEM). (D) Lysates of SW480 and SW620 cells were co‐immunoprecipitated with anti‐Cx26 or anti‐PIMT antibodies, and then PIMT and Cx26 protein levels were detected by immunoblot. IgG was used as a loading control. (E) Immunofluorescence of Cx26 (green) and PIMT (red) in SW480 and SW620 cells. Nuclei were stained with DAPI (blue). Scale bar: 10 µm. (F) SW480 cells were treated with TGF‐β for 0, 24, and 48 h; cell lysates were collected for co‐immunoprecipitation with anti‐Cx26 antibody. IgG was used as a loading control. (G) Western blot of the protein level of Cx26 in the cytoplasm and plasma membrane in SW620 cells transfected with shRNA‐NC or shRNA‐PIMT. Na, K‐ATPase and Tubulin were measured as loading control of membrane and cytoplasm, respectively. (H) Immunofluorescence of Cx26 (green) in SW620 cells transfected with or without shRNA‐PIMT. Mitochondria were stained with MitoTracker Red (red). Nuclei were stained with DAPI (blue). Scale bar, 50 µm. (I) Immunoblot of Cx26, PIMT, COX4, and Tubulin in the cytoplasmic and mitochondria‐enriched fractions of SW620 cells transfected with or without shRNA‐PIMT. Tubulin and COX4 were used as markers and loading controls for the cytoplasmic and mitochondria‐enriched fractions, respectively. ^***^ *p* < 0.001 *vs*. SW620 cells transfected without shRNA‐PIMT; by unpaired student's *t*‐test (mean ± SEM).

To explore whether the interaction between PIMT and Cx26 affects the translocation of Cx26, we detected the protein level of Cx26 in the cytoplasm and cell membrane of SW620 cells transfected with or without shRNA‐PIMT. Interestingly, the results showed an increased translocation of Cx26 from the cytoplasm to the cell membrane in SW620‐shRNA‐PIMT cells (Figure 2G). Compared with SW620‐shRNA‐NC cells, the gap junction function of SW620‐shRNA‐PIMT cells was restored (Figure S7B,C). At the same time, knockdown of PIMT reduced the mitochondrial localization of Cx26 in SW620 cells (Figure 2H,I). These data demonstrate that PIMT can promote the mitochondrial translocation of Cx26, thereby disrupting gap junctions in cells.

### 3.3 PIMT Methylates Asparagine 176 of Cx26 for Mitochondrial Translocation

PIMT is a relatively conserved methyltransferase that is widely expressed in various cells and can repair heterogeneous aspartic acid by methylation and maintain an accurate protein configuration. Given that PIMT is a methyltransferase, we sought to determine whether Cx26 is a substrate of PIMT. To this end, we mutated glycine 88 of PIMT to alanine (PIMT‐G88A). According to literature reports, the G88 site of PIMT plays a key role in its enzymatic activity and is the key site for PIMT to bind to the substrate. The results of the co‐IP experiment showed that wild‐type PIMT can bind to Cx26, while PIMT‐G88A cannot bind to Cx26 (Figure 3A), indicating that Cx26 binds to the substrate‐binding region of PIMT. Considering that PIMT plays a role in protein repair by recognizing and converting D‐aspartyl and L‐isoaspartyl residues resulting from spontaneous deamidation back to the normal L‐aspartyl form, we examined whether PIMT methylates Cx26 and sought to identify the modified sites of Cx26. We enriched the Cx26 protein in cell lysates by IP and identified the methylation and deamidation of asparagine 176 of Cx26 by nano‐LC‐ESI‐MS/MS analysis (Figure 3B–D). Consistently, the mutation of asparagine 176 of Cx26 reduced Cx26 levels in the mitochondria‐enriched fraction while increasing its levels in the cytoplasmic fraction (Figure 3E,F). These data demonstrate that PIMT methylates Cx26 at asparagine 176, which can influence the localization of Cx26.

**FIGURE 3:**
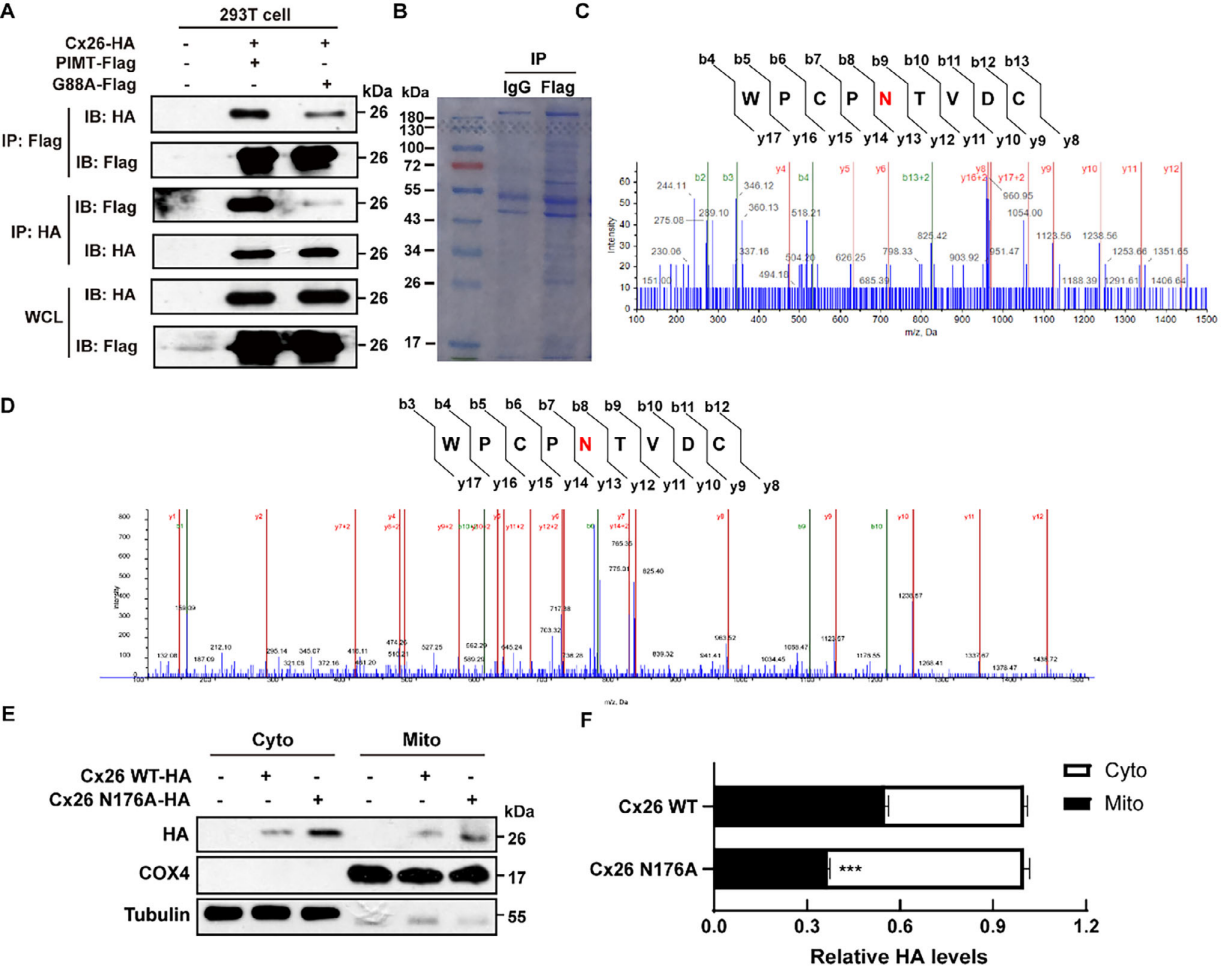
PIMT methylates asparagine 176 of Cx26 for mitochondrial translocation. (A) HEK293T cells were co‐transfected with Cx26 ‐ HA and either PIMT‐WT‐Flag or PIMT‐G88A‐Flag. Flag or HA was immunoprecipitated from cell lysates by immunoblotting with different antibodies. (B) The lysate of HEK293T cells transfected with the Cx26‐Flag plasmid was immunoprecipitated with IgG or Flag antibody, and the gel was stained with Coomassie blue after electrophoresis. (C,D) LC‐MS analysis of Cx26. (E,F) Immunoblot and statistical analysis of HA, COX4, and Tubulin in cytoplasmic and mitochondria‐enriched fractions of HEK293T cells transfected with Cx26 WT‐HA or Cx26 N176A‐HA plasmid. Tubulin and COX4 were used as markers and loading controls for the cytoplasmic and mitochondria‐enriched fractions, respectively. ^***^ *p* < 0.001 vs. Cx26‐WT; by unpaired student's *t*‐test (mean ± SEM).

### 3.4 PIMT Increases the Stability of Cx26

Deamidation of asparagine is a spontaneous posttranslational modification of proteins that disrupts protein structure and function and affects protein stability. Therefore, we continued to explore the effect of PIMT on the stability of the Cx26 protein. We overexpressed the Cx26 WT‐HA or Cx26 N176A‐HA plasmid in 293T cells and treated them with cycloheximide (CHX) for 0, 3, 6, 12, and 24 h. Western blot analysis showed that the protein half‐life of Cx26‐N176A was shorter than that of Cx26‐WT (Figure 4A–C), indicating that the presence of asparagine at position 176 contributes to Cx26 protein stability. PIMT overexpression increased the amount of Cx26‐WT protein remaining after CHX treatment, but had no significant effect on the stability of Cx26‐N176A (Figure 4D,E). These findings suggest that PIMT‐mediated modification of Cx26 at N176 may contribute to Cx26 protein stabilization. Similarly, SW620 cells showed a longer protein half‐life of Cx26 than SW480 cells (Figure S8), which is related to the stronger interaction of Cx26 and PIMT. The proteasome inhibitor MG132 reversed the degradation of Cx26 caused by PIMT knockdown and CHX treatment, indicating that Cx26 is mainly degraded by the ubiquitin‐proteasome system (Figure 4F,G). Ubiquitination of Cx26 was also verified in co‐IP experiments in 293T cells overexpressing Cx26‐Flag and Ub‐HA plasmids (Figure 4H). Compared with SW620 cells, the ubiquitination‐dependent degradation of Cx26 was stronger in SW480 cells (Figure 4I). The ubiquitination of Cx26 was lower in 293T cells transfected with Cx26‐HA, Ub‐His, and PIMT WT‐Flag than in those without PIMT WT‐Flag. When PIMT G88A‐Flag was used to replace PIMT WT‐Flag, the ubiquitination of Cx26 was rescued (Figure 4J,K). These results suggest that PIMT can inhibit the ubiquitination of Cx26 and increase its protein stability.

**FIGURE 4:**
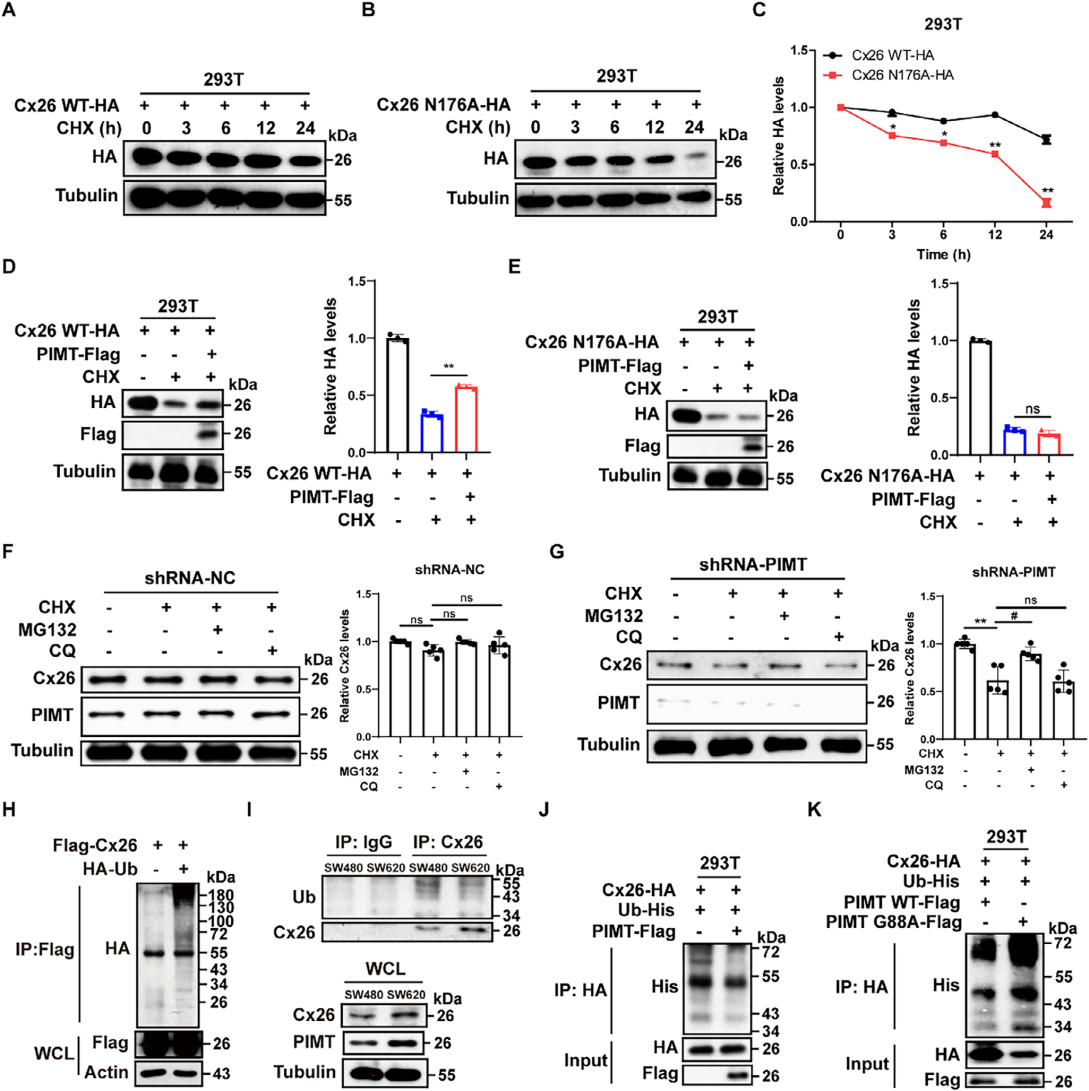
PIMT increases the stability of Cx26. (A–C) Immunoblot and statistical analysis of HA in HEK293T cells transfected with Cx26 WT‐HA or Cx26 N176A‐HA plasmid after CHX (100 µM) treatment for 0, 3, 6, 12, and 24 h. Tubulin was used as a loading control. ^*^ *p* < 0.05, ^**^ *p* < 0.01 vs. Cx26 WT‐HA; by two‑way ANOVA followed by Šídák's multiple‑comparisons test (mean ± SEM). (D) Immunoblot and statistical analysis of HA in HEK293T cells transfected with Cx26 WT‐HA or PIMT‐Flag and Cx26 WT‐HA after CHX (100 µM) treatment for 24 h. Tubulin was used as a loading control. ^**^ *p* < 0.01 *vs*. Cx26 WT‐HA+CHX; by one‐way ANOVA followed by Šídák's multiple comparisons test (mean ± SEM). (E) Immunoblot and statistical analysis of HA in HEK293T cells transfected with Cx26 N176A‐HA or PIMT‐Flag and Cx26 N176A‐HA after CHX (100 µM) treatment for 24 h. Tubulin was used as a loading control. Statistical significance was determined by one‐way ANOVA followed by Šídák's multiple comparisons test (mean ± SEM). ns means no significant difference. (F‐G) SW620‐shRNA‐NC or SW620‐shRNA‐PIMT cells were treated with MG132 (2 µM) or CQ (100 µM) and CHX (100 µM) for 24 h. The protein levels of Cx26 and PIMT were analyzed by Western blot and statistical analysis. Tubulin was used as a loading control. ns means no significant difference, ^**^ *p* < 0.01 *vs*. Control, ^#^ *p* < 0.05 *vs*. CHX; by one‐way ANOVA followed by Šídák's multiple comparisons test (mean ± SEM). (H) Lysates of HEK293T transfected with Cx26‐Flag or Cx26‐Flag and Ub‐HA were co‐immunoprecipitated with a Flag antibody, and then HA, Flag, and Actin protein levels were detected by immunoblot. Actin was used as a loading control. (I) Lysates of SW480 and SW620 cells were co‐immunoprecipitated with IgG or Cx26 antibody, and then Ub, Cx26, PIMT, and Tubulin protein levels were detected by immunoblot. Tubulin was used as a loading control. (J) Lysates of HEK293T cells transfected with Cx26‐HA and Ub‐His or Cx26‐HA, Ub‐His, and PIMT‐Flag were co‐immunoprecipitated with HA antibody, and then His, HA, and Flag protein levels were detected by immunoblot. (K) Lysates of HEK293T cells transfected with Cx26‐HA, Ub‐His, and PIMT WT‐Flag or PIMT G88A‐Flag were co‐immunoprecipitated with HA antibody, and then His, HA, and Flag protein levels were detected by immunoblot.

### 3.5 Mitochondrial Cx26 and Its Related Ca^2+^ Uptake Control the EMT Ability for CRC Growth and Metastasis

To explore the function of mitochondrial‐localized Cx26, we examined the effect of Cx26 on mitochondrial Ca^2+^ uptake. We detected mitochondrial Ca^2+^ levels by monitoring the fluorescence intensity of pCMV‐CEPIA4‐mit plasmids. Compared with SW480‐NC cells, SW480‐OE‐Cx26 cells had significantly increased mitochondrial Ca^2+^ uptake (Figure 5A). This result was also verified in HEK293T cells (Figure S9A). However, the N176A mutation of Cx26 significantly reduced the mitochondrial uptake of Ca^2+^, indicating that mitochondrial translocation of Cx26 is important for mitochondrial Ca^2+^ uptake (Figure 5B). A large amount of Ca^2+^ is stored in the endoplasmic reticulum (ER), so we further explored the effect of Cx26 on ER‐mitochondria crosstalk. Electron microscopy and co‐IP showed that Cx26 mediates ER‐mitochondria crosstalk via IP3R‐I/II/III and VDAC1 (Figure 5C–F).

**FIGURE 5:**
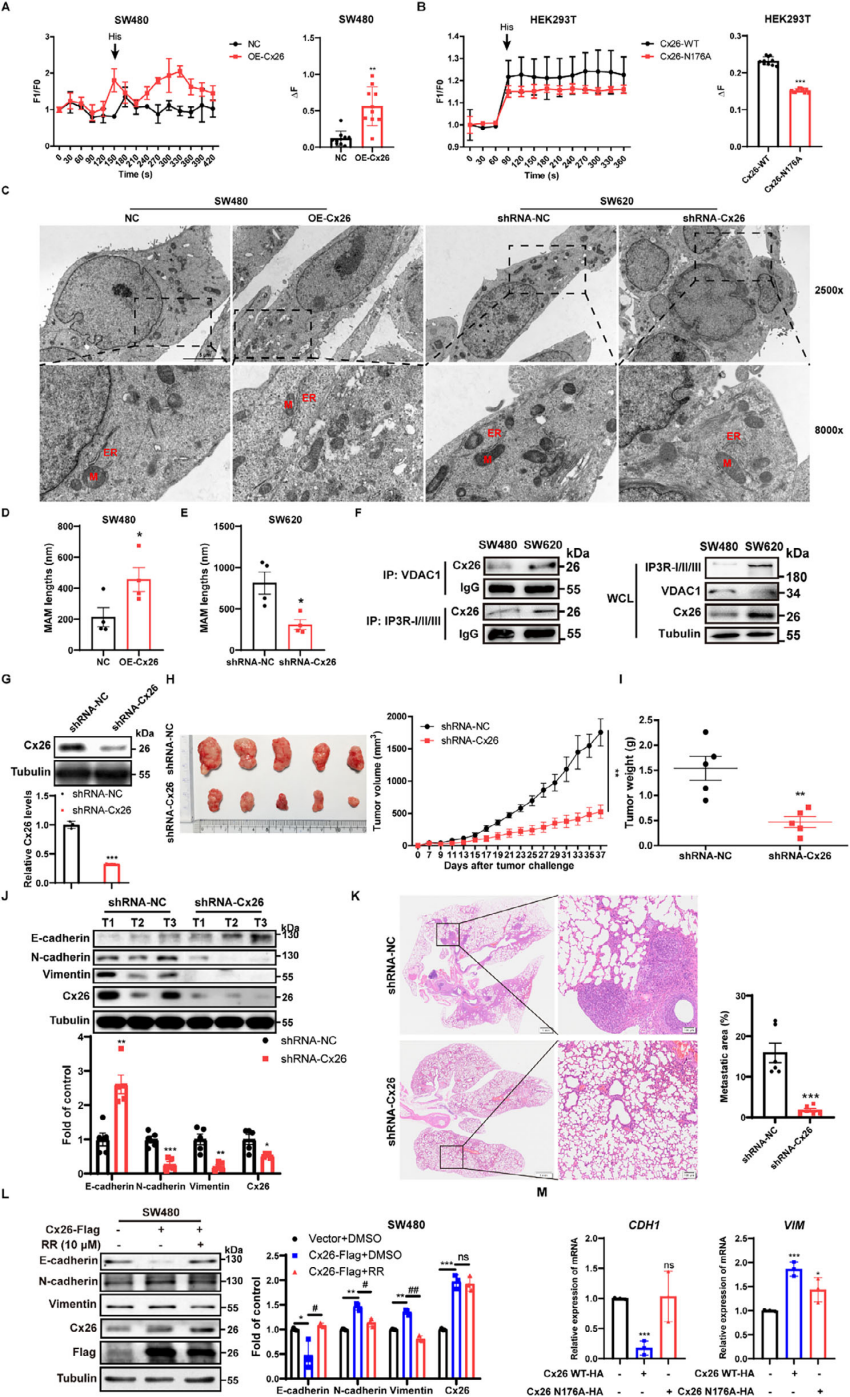
Mitochondrial Cx26 and its related Ca^2+^ uptake control the EMT ability for CRC growth and metastasis. (A) Representative curves and statistical analysis of mitochondrial Ca^2+^ before and after histamine (20 µM) treatment in SW480‐pCMV‐CEPIA4‐mit cells transfected with or without Cx26‐HA plasmid. ^**^ *p* < 0.01 *vs*. NC; by unpaired student's *t*‐test (mean ± SEM). (B) Representative curves and statistical analysis of mitochondrial Ca^2+^ before and after histamine (20 µM) treatment in HEK293T cells transfected with pCMV‐CEPIA4‐mit and Cx26‐WT or Cx26‐N176A. ^***^ *p* < 0.001 *vs*. Cx26‐WT; by unpaired student's *t*‐test (mean ± SEM). (C) Representative electron micrographs of SW480‐NC, SW480‐OE‐Cx26, SW620‐shRNA‐NC and SW620‐shRNA‐Cx26 cells. ER, endoplasmic reticulum. M, mitochondrion. Scale bar, 5 µm. (D‐E) Average length of mitochondria‐associated endoplasmic reticulum membranes (MAM) in SW480‐NC, SW480‐OE‐Cx26, SW620‐shRNA‐NC and SW620‐shRNA‐Cx26 cells. ^*^ *p* < 0.05 *vs*. NC or shRNA‐NC; by unpaired student's *t*‐test (mean ± SEM). (F) Immunoblot analysis of Cx26 in the IP3R‐I/II/III or VDAC1 immunoprecipitates of total lysates. WCL, whole‐cell lysate. (G) The expression levels of Cx26 and Tubulin in SW620‐shRNA‐NC and SW620‐shRNA‐Cx26 cells were analyzed by western blot, and Tubulin was used as a loading control. ^***^ *p* < 0.001 *vs*. shRNA‐NC; by unpaired student's *t*‐test (mean ± SEM). (H) Representative images and volume growth curves of tumors established by SW620‐shRNA‐NC and SW620‐shRNA‐Cx26 cells (*n* = 5). ^**^ *p* < 0.01 *vs*. shRNA‐NC; by two‐way repeated‐measures ANOVA with Geisser‐Greenhouse correction (mean ± SEM). (I) Weight of tumors established by SW620‐shRNA‐NC and SW620‐shRNA‐Cx26 cells (*n* = 5). ^**^ *p* < 0.01 *vs*. shRNA‐NC; by unpaired student's *t*‐test (mean ± SEM). (J) The expression levels of N‐cadherin, E‐cadherin, Vimentin, Cx26, and Tubulin in tumors established by SW620‐shRNA‐NC and SW620‐shRNA‐Cx26 cells were analyzed by western blot and statistical analysis, and Tubulin was used as a loading control (*n* = 5). ^*^ *p* < 0.05, ^**^ *p* < 0.01, ^***^ *p* < 0.001 *vs*. shRNA‐NC; by unpaired student's *t*‐test (mean ± SEM). (K) Representative H&E‐stained sections of lung tissues from mice intravenously injected with SW620 cells expressing shRNA‐NC or shRNA‐Cx26. The metastatic area was quantified using QuPath (*n* = 6). ^***^ *p* < 0.001 *vs*. shRNA‐NC; by unpaired student's *t*‐test (mean ± SEM). (L) SW480 cells were transfected with Cx26‐Flag plasmid, and then treated with RR (10 µM) for 24 h. The protein levels of E‐cadherin, N‐cadherin, Vimentin, Cx26, Flag, and Tubulin were analyzed by western blot and statistical analysis, and Tubulin were performed as loading controls, respectively. Relative E‐cadherin, N‐cadherin, Vimentin, and Cx26 levels were normalized to Tubulin. ns means no significant difference, ^*^ *p* < 0.05, ^**^ *p* < 0.01, ^***^ *p* < 0.001 *vs*. Vector+DMSO, ^#^ *p* < 0.05, ^##^ *p* < 0.01 *vs*. Cx26‐Flag+DMSO; by one‐way ANOVA followed by Šídák's multiple comparisons test (mean ± SEM). (M) The mRNA levels of *CDH1* and *VIM* were detected in SW480 cells transfected with Cx26 WT‐HA or Cx26 N176A‐HA plasmids. Data were normalized to *GAPDH*. ns means no significant difference, ^*^ *p* < 0.05, ^***^ *p* < 0.001 *vs*. Control; by one‐way ANOVA followed by Dunnett's multiple comparisons test (mean ± SEM).

Then, we found that overexpression of Cx26 accelerated EMT in SW480 cells, whereas knockdown of Cx26 reversed EMT in SW620 cells (Figure S9B,C). In vivo experiments showed that Cx26 knockdown in SW620 cells significantly suppressed subcutaneous xenograft tumor growth and reduced EMT levels in tumor tissues. Moreover, Cx26 knockdown markedly reduced pulmonary metastatic burden in the tail vein injection‐induced experimental metastasis model (Figure 5G–K). Conversely, Cx26 overexpression in SW480 cells significantly promoted subcutaneous xenograft tumor growth and enhanced the EMT phenotype in tumor tissues, while markedly increasing pulmonary metastatic burden in the tail vein injection‐induced experimental metastasis model (Figure S10). Consistent with these findings, Cx26 knockdown in B16F10 cells significantly suppressed subcutaneous xenograft tumor growth and reduced EMT levels in tumor tissues, and also markedly decreased pulmonary metastatic burden in the tail vein injection‐induced experimental metastasis model (Figure S11).

To explore the relationship between the effect of Cx26 on mitochondrial Ca^2+^ uptake and EMT, we independently inhibited mitochondrial Ca^2+^ uptake in SW620 cells using either ruthenium red (RR) or MCU‐i4. Both treatments reduced the expression levels of the EMT markers N‐cadherin and Vimentin, increased E‐cadherin expression, and suppressed the EMT phenotype (Figure S12A,B). Furthermore, treatment of SW480‐OE‐Cx26 cells with RR reversed Cx26‐induced EMT, demonstrating that Cx26 enhances EMT by promoting mitochondrial Ca^2+^ uptake (Figure 5L). Meanwhile, compared with SW480 cells, the mRNA levels of the EMT marker proteins E‐cadherin and Vimentin were significantly decreased and increased in SW480‐OE‐Cx26‐WT cells but not in SW480‐OE‐Cx26‐N176A cells, indicating that impairing the mitochondrial localization of Cx26 can disrupt the pro‐EMT effect of Cx26 (Figure 5M and Figure S12C). These results demonstrate the important role of mitochondrial translocation of Cx26 in tumor growth and EMT in CRC, which is correlated with mitochondrial Ca^2+^ uptake.

### 3.6 Highly Malignant CRC Is More Sensitive to bortezomib

More malignant cancers are usually more resistant to current anticancer therapies and are more difficult to treat. Considering that the mitochondrial translocation of Cx26 contributes to the malignant progression of CRC, we asked whether *GJB2* expression could predict sensitivity to clinically relevant drugs. We therefore analyzed the correlation between *GJB2* expression and drug sensitivity across 23 CRC cell lines in the GDSC1 and GDSC2 datasets, using IC_50_ values as the sensitivity metric. Higher *GJB2* expression was generally associated with reduced sensitivity to most chemotherapeutic agents. In contrast, bortezomib (BTZ), a 26S proteasome inhibitor and first‐line therapy for multiple myeloma and mantle cell lymphoma, showed an inverse correlation, with higher *GJB2* expression corresponding to lower IC_50_ values (Figure 6A). This unexpected association prompted us to investigate whether BTZ preferentially suppresses Cx26‐high CRC cells. We then compared BTZ and commonly used drugs for the clinical treatment of CRC in their ability to kill primary SW480 and metastatic SW620 cells. SW620 cells were less sensitive or had no significant difference from SW480 cells to 5‐fluorouracil, oxaliplatin, irinotecan, paclitaxel, and etoposide. Only BTZ showed stronger efficacy against the proliferation of SW620 cells than SW480 cells (Figure S13). Consistent with these findings, BTZ also exerted a stronger inhibitory effect on the proliferation of highly metastatic B16F10 cells than on that of B16F1 cells (Figure S14).

**FIGURE 6:**
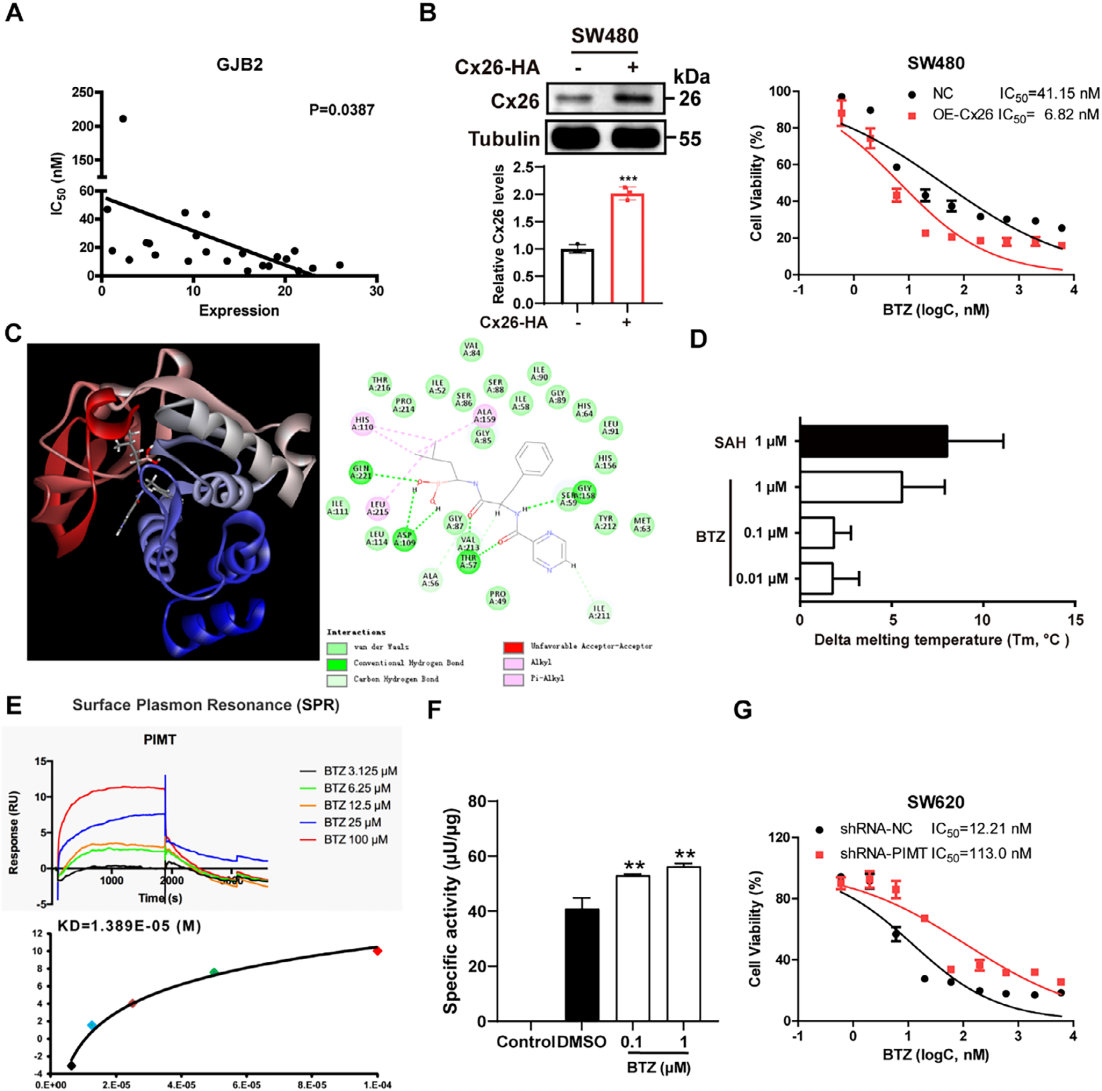
Highly malignant CRC is more sensitive to bortezomib. (A) The relation between *GJB2* expression and IC_50_ values of BTZ in CRC cells was analyzed by GDSC. (B) The protein levels of Cx26 and Tubulin in SW480‐NC and SW480‐OE‐Cx26 cells were detected by western blot. Tubulin was used as a loading control. The IC_50_ values of BTZ in SW480‐NC and SW480‐OE‐Cx26 cells were measured by MTT assay. ^***^ *p* < 0.001 *vs*. Control; by unpaired student's *t*‐test (mean ± SEM). (C) Molecular docking analysis of BTZ and PIMT via Discovery Studio. On the right is the 2D diagram of the interaction between BTZ and amino acid residues of PIMT. (D) PIMT was incubated with DMSO, BTZ (0.01, 0.1, 1 µM), and SAH (1 µM), respectively, delta Tm was calculated compared to the DMSO group. Data are presented as mean ± SEM of at least three replicates. (E) Dose‐response curves for a gradient dilution of BTZ binding to PIMT were measured by Biacore T200. (F) PIMT activity was determined using a methyltransferase activity assay kit. ^**^ *p* < 0.01 *vs*. DMSO; by one‐way ANOVA followed by Dunnett's multiple comparisons test (mean ± SEM). (G) The IC_50_ values of BTZ in SW620‐shRNA‐NC and SW620‐shRNA‐PIMT cells were measured by MTT assay. Data are presented as the mean ± SEM of three replicates.

BTZ significantly inhibited the proliferation of CRC cells, attenuated their EMT phenotype, and suppressed their invasive capacity, with stronger inhibitory effects observed in cells with higher metastatic potential (Figures S15 and S16). Furthermore, we confirmed that Cx26 overexpression increased the sensitivity of SW480 cells to BTZ (Figure 6B). Notably, the differential sensitivity of SW480 and SW620 cells to BTZ could not be explained solely by proteasome inhibition (Figure S17).

Cellular thermal shift assays (CETSAs) and molecular docking assays showed that BTZ did not interact with Cx26 (PDB ID: 5ERA) (Figure S18A,B). Interestingly, the molecular docking assay predicted that BTZ binds to PIMT (PDB ID: 1KR5, ‐CDOCKER INTERACTION ENERGY = 68.0211), which was higher than that of BTZ and the positive control proteasome (PDB ID: 5LE5, ‐CDOCKER INTERACTION ENERGY = 46.5801) (Figure 6C and Figure S18C). This indicates that PIMT might be a potential target of BTZ. To test our speculation, CETSA was used to assess the interaction of PIMT and BTZ. As shown in Figure S18D,E, BTZ could interact with PIMT and enhance the thermostability of PIMT. In addition, differential scanning fluorimetry (DSF) analysis was used to determine the interaction between PIMT and BTZ by measuring the melting temperature (Tm) of PIMT. PIMT, as a methyltransferase, can specifically transfer the methyl group from the methyl donor S‐adenosyl‐L‐methionine (SAM) to the heterogeneous aspartic acid residues that require methylation repair. After SAM loses its methyl group, S‐adenosyl‐L‐homocysteine (SAH) is generated, and SAH can bind to the methyl donor binding region of PIMT. Hence, we used SAH as a positive control to evaluate the interaction between PIMT and BTZ via DSF. As shown in Figure 6D, the Tm of PIMT was increased by 5.6°C and 8°C after incubation with BTZ (1 µM) and SAH (1 µM), respectively, suggesting that BTZ could directly bind to PIMT and promote the stability of PIMT. These results were further confirmed by surface plasmon resonance (SPR), which detected the direct interaction of PIMT and BTZ (Figure 6E). In an in vitro methyltransferase activity assay, we found that BTZ enhanced the enzymatic activity of PIMT (Figure 6F). Furthermore, PIMT knockdown reduced the sensitivity of SW620 cells to BTZ (Figure 6G). These results suggest that BTZ may exert anti‐tumor effects against highly malignant CRC by targeting the PIMT‐Cx26 interaction, which represents a mechanism distinct from that of most currently used anticancer drugs.

### 3.7 PIMT‐regulated Mitochondrial Localization of Cx26 and Ca^2+^‐ROS‐ERK Signaling Are Critical for the Efficacy of BTZ Against Highly Malignant CRC

We speculated that the positive correlation between high expression of Cx26 in CRC cells and BTZ sensitivity may be regulated by PIMT. Co‐IP and immunoblotting experiments showed that BTZ can increase the interaction between PIMT and Cx26 in SW620 cells (Figure 7A). Concurrently, mitochondria‐enriched and cytoplasmic fractions were isolated from SW620‐shRNA‐NC and SW620‐shRNA‐PIMT cells with or without BTZ treatment. Western blot analysis showed that BTZ significantly increased Cx26 levels in the mitochondria‐enriched fraction of SW620‐shRNA‐NC cells, whereas this increase was markedly attenuated by PIMT knockdown (Figure 7B,C). These results suggest that BTZ can increase the mitochondrial translocation of Cx26 by promoting the interaction between PIMT and Cx26.

**FIGURE 7:**
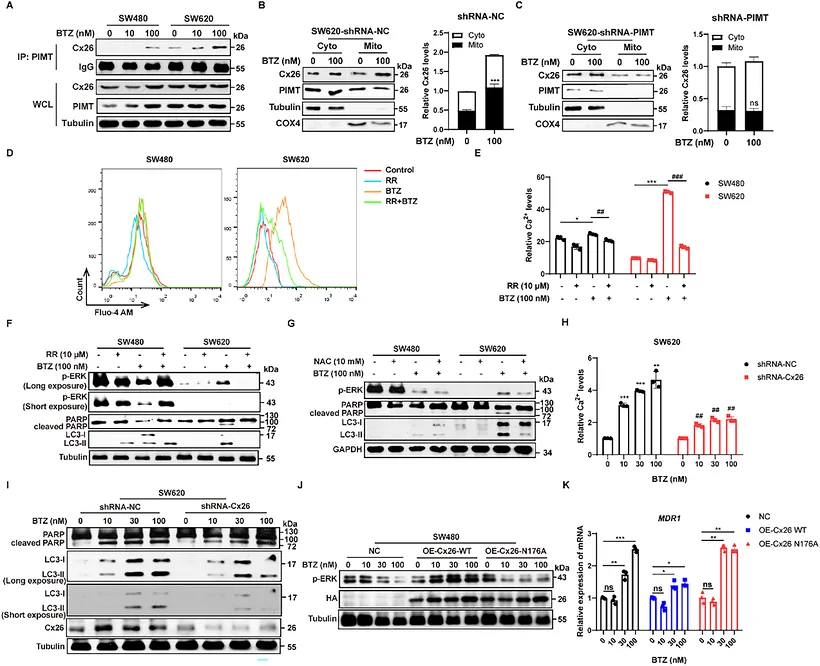
PIMT‐regulated mitochondrial localization of Cx26 and Ca^2+^‐ROS‐ERK signaling are critical for the efficacy of BTZ against highly malignant CRC. (A) SW480 and SW620 cells were treated with BTZ (0, 10, 100 nM) for 18 h; an anti‐PIMT antibody was used for co‐immunoprecipitation, and the expression of Cx26 and PIMT was determined by western blot. (B‐C) Immunoblot of Cx26, PIMT, Tubulin, and COX4 in cytoplasmic and mitochondria‐enriched fractions of SW620‐shRNA‐NC or SW620‐shRNA‐PIMT cells stimulated with or without BTZ (100 nM) for 24 h. Tubulin and COX4 were used as markers and loading controls for the cytoplasmic and mitochondria‐enriched fractions, respectively. ns means no significant difference, ^***^ *p* < 0.001 *vs*. 0 nM; by unpaired student's *t*‐test (mean ± SEM). (D‐E) Flow cytometry analysis of intracellular Ca^2+^ by Fluo‐4 AM staining in SW480 and SW620 cells treated with RR (10 µM), BTZ (100 nM), or RR+BTZ for 24 h. ^*^ *p* < 0.05, ^***^ *p* < 0.001 *vs*. SW480 or SW620, ^##^ *p* < 0.01, ^###^ *p* < 0.001 *vs*. SW480+BTZ or SW620+BTZ; by two‑way ANOVA followed by Šídák's multiple‑comparisons test (mean ± SEM). (F) SW480 and SW620 cells were treated with RR (10 µM), BTZ (100 nM), or RR+BTZ for 24 h. The protein levels of p‐ERK, PARP, cleaved PARP, LC3‐I, LC3‐II, and Tubulin were detected by western blot, and Tubulin was used as a loading control. (G) The protein levels of p‐ERK, PARP, cleaved PARP, LC3‐I, LC3‐II, and GAPDH were detected by western blot, and GAPDH was used as a loading control. The results shown are representative of three replicates. (H) Flow cytometry analysis of intracellular Ca^2+^ by Fluo‐4 AM staining in SW620‐shRNA‐NC and SW620‐shRNA‐Cx26 cells treated with BTZ (0, 10, 30, 100 nM) for 24 h. ^**^ *p* < 0.01, ^***^ *p* < 0.001 *vs*. shRNA‐NC (BTZ 0 nM), ^##^ *p* < 0.01 *vs*. shRNA‐Cx26 (BTZ 0 nM); by two‑way ANOVA followed by Dunnett's multiple comparisons test (mean ± SEM). (I) Immunoblot of PARP, cleaved PARP, LC3‐I, LC3‐II, Cx26 and Tubulin in SW620‐shRNA‐NC and SW620‐shRNA‐Cx26 cells treated with BTZ (0, 10, 30, 100 nM) for 24 h. (J) SW480‐NC, SW480‐OE‐Cx26‐WT, and SW480‐OE‐Cx26‐N176A cells were treated with BTZ (0, 10, 30, 100 nM) for 24 h. Immunoblotting of p‐ERK, HA, and Tubulin was used as a loading control. (K) SW480‐NC, SW480‐OE‐Cx26‐WT and SW480‐OE‐Cx26‐N176A cells were treated with BTZ (0, 10, 30, 100 nM) for 24 h. The mRNA levels of *MDR1* were detected by QPCR, and data were normalized to *GAPDH*. ns means no significant difference, ^*^ *p* < 0.05, ^**^ *p* < 0.01, ^***^ *p* < 0.001 *vs*. NC, OE‐Cx26 WT, or OE‐Cx26 N176A (BTZ 0 nM); by two‑way ANOVA followed by Dunnett's multiple comparisons test (mean ± SEM).

In addition, BTZ treatment significantly increased the intracellular Ca^2+^ level of SW620 cells compared with SW480 cells (Figure S19). When we treated SW480 and SW620 cells with RR and/or BTZ, the results of flow cytometry showed that, compared with SW480 cells, RR can significantly reverse the massive increase in intracellular Ca^2+^ levels in BTZ‐incubated SW620 cells (Figure 7D,E). Through gene set enrichment analysis (GSEA), we found that Cx26 was negatively correlated with the MAPK pathway activity (Figure S20). It should be noted that this negative association reflects the basal physiological state of untreated CRC cells. Consistent with this finding, SW620 cells, which exhibited higher Cx26 expression, showed lower basal p‐ERK levels than SW480 cells. However, following BTZ treatment, Cx26 protein levels were further increased above the basal level in SW620 cells, coinciding with a marked increase in p‐ERK levels. These findings suggest that the substantial increase in Cx26 following BTZ treatment shifts ERK signaling from a basal state to an acute stress response. Accordingly, BTZ treatment elicited distinct acute ERK responses in the two cell lines: BTZ reduced p‐ERK levels in SW480 cells, which exhibited relatively low basal Cx26 expression, but increased p‐ERK levels in SW620 cells, which exhibited high basal Cx26 expression (Figure S21). Moreover, BTZ exhibited more significant potential to induce apoptosis and autophagy in SW620 cells (Figure S22). Similarly, treatment with the ERK inhibitor PD0325901 significantly reduced the sensitivity of SW620 cells to BTZ and inhibited BTZ‐induced autophagy and apoptosis in SW620 cells (Figure S23). Then, we found that RR treatment of SW620 cells could significantly inhibit the BTZ‐induced increase in p‐ERK levels and inhibit the cleavage of PARP and the production of LC3‐II, suggesting that inhibition of mitochondrial Ca^2+^ uptake could inhibit BTZ‐induced autophagy and apoptosis in SW620 cells, and that the regulation of ERK signaling by BTZ in SW620 cells was affected by Ca^2+^ (Figure 7F).

Excessive mitochondrial Ca^2+^ uptake leads to reactive oxygen species (ROS) production. Compared with SW480 cells, BTZ produced more ROS in SW620 cells, and inhibition of mitochondrial Ca^2+^ uptake significantly reduced ROS production. However, the ROS scavenger NAC had no effect on the BTZ‐induced increase in intracellular Ca^2+^ levels, further demonstrating that increased mitochondrial Ca^2+^ uptake stimulated ROS production (Figure S24). Moreover, scavenging ROS significantly inhibited the apoptosis of SW620 cells, significantly reduced activation of the ERK pathway, and inhibited PARP cleavage and LC3‐II production compared with SW480 cells (Figure 7G and Figure S25). When we knocked down Cx26 in SW620 cells, the magnitude of the BTZ‐induced Ca^2+^ increase was reduced, as was the extent of PARP cleavage and elevated LC3‐II protein levels (Figure 7H,I). In SW480 cells, overexpression of Cx26‐WT but not Cx26‐N176A activated p‐ERK by BTZ and inhibited BTZ‐induced tumor resistance (Figure 7J,K). These results indicate that BTZ induces apoptosis and autophagy in highly malignant metastatic CRC cells by increasing the mitochondrial translocation of Cx26 and activating the Ca^2+^‐ROS‐ERK pathway.

## 4 Discussion

Metastasis is the leading cause of death in CRC patients, and CRC after metastasis is resistant to drugs and difficult to treat. A study analyzed the expression levels of Cx26 in colorectal nontumor tissue, primary tumor tissue, and lung metastases in 106 male and 61 female CRC patients and found that the level of Cx26 in lung metastases was significantly higher than that in primary CRC. It is worth noting that Cx26 was mainly expressed on the plasma membrane in normal epithelium and adenoma tissues. While Ezumi et al. first reported cytoplasmic accumulation of Cx26 in CRC metastases and associated it with poor prognosis [13], our work provides the first evidence that Cx26's specific mitochondrial translocation, which is regulated by PIMT‐dependent methylation, actively promotes EMT, Ca^2+^ signaling, and metastatic progression. This discovery shifts the paradigm from viewing Cx26 mislocalization as a passive marker of aggressiveness to recognizing it as a functional driver of CRC malignancy through organelle‐specific signaling (Figure 1).

The expression and function of many molecules change during tumor malignancy, and EMT is the key to tumor invasion and metastasis. We found that the mitochondrial localization of Cx26 promotes mitochondrial Ca^2+^ uptake and ER‐mitochondria crosstalk via IP3R‐I/II/III and VDAC1, thereby increasing the EMT level and metastatic capacity of CRC (Figure 5). However, the mitochondrial translocation of Cx26 is not affected by its amino acids at positions 1–40 (predicted signal peptides) but by its interacting protein PIMT (Figure 2 and Figure S4). In extracellular loop 2 of Cx26, K168, D179, and the main‐chain carbonyl groups of T177 and N176 form hydrogen bonds and salt bridges with the opposite connexin in the opposing hemichannel, and these interactions contribute to the connection of intercellular hemichannels [23, 24]. In the human connexin protein family, D179 is conserved, but K168, N176, and T177 differ. Cx46 N188 and Cx32 N175 mutations cannot form functional homotypic gap junction channels, which correspond to Cx26 N176, but the effect of Cx26 N176 on its function is not clear [25]. We found that the normal conformation of Cx26 N176 is important for its localization and stability (Figures 3 and 4). The Cx26 N176 mutation could reduce its mitochondrial localization, thereby inhibiting mitochondrial Ca^2+^ uptake and disrupting its pro‐EMT function (Figures 3 and 5).

Asparagine undergoes deamidation, a spontaneous posttranslational modification of proteins that produces isoaspartate (isoAsp) residues, which can form twists in the protein backbone, disrupt the structure and function of proteins, and affect protein stability [26]. PIMT can use S‐adenosylmethionine (SAM) as a methyl donor to specifically methylate and repair isoAsp residues, maintaining normal protein configuration and function [17, 27]. Figure 2 demonstrates that the higher the degree of malignancy is, the stronger the interaction between Cx26 and PIMT in CRC, and PIMT can promote Cx26 translocation to mitochondria. SAM supplementation in primary hepatocyte culture can increase the protein expression of Cx26, but it cannot induce Cx26 localization to the cell membrane, nor does it improve the communication function of intercellular gap junctions, indicating that SAM supplementation is beneficial for improving the protein level of Cx26, but the membrane localization of Cx26 may be regulated by other factors [28]. Approximately 30% of SAM is found in mitochondria, where it can be converted to S‐adenosyl‐L‐homocysteine (SAH) in methylation reactions of proteins, RNA, and DNA and re‐enters the methionine cycle [29, 30]. These studies suggest that the utilization of SAM may be the reason why Cx26 repaired by PIMT shows preferential mitochondrial localization.

Mitochondria can use ATP to provide energy to cells and are important intracellular Ca^2+^ storage sites [31]. Intracellular Ca^2+^ controls many functions, such as adhesion, proliferation, EMT, metabolism, life and death of tumors. Appropriate mitochondrial Ca^2+^ uptake is beneficial for the regulation of cell metabolism and bioenergetics, while excessive mitochondrial Ca^2+^ uptake induces cell death, so maintaining mitochondrial homeostasis is essential for cell survival [32]. Given that mitochondrial Cx26 is closely related to the degree of malignancy of CRC and its function in regulating mitochondrial Ca^2+^ uptake, we sought to find a treatment for highly malignant metastatic CRC. Excitingly, unlike commonly used first‐line chemotherapy drugs for CRC, we found that BTZ is more effective in killing highly malignant metastatic CRC. The finding that BTZ selectively targets metastatic CRC represents a striking departure from its conventional application in hematologic malignancies. While BTZ is a mainstay therapy for multiple myeloma through proteasome inhibition, we demonstrate that its efficacy against Cx26‐high metastatic CRC stems from an entirely distinct mechanism: direct binding to PIMT, amplification of mitochondrial Cx26 localization, and catastrophic activation of the Ca^2^^+^‐ROS‐ERK pathway (Figures 6 and 7). This unexpected vulnerability of metastatic CRC to BTZ challenges the prevailing assumptions that solid tumors are inherently resistant to proteasome inhibitors. Our data instead position BTZ as a molecular tool to exploit mitochondrial Cx26 addiction in advanced CRC.

In conclusion, our study for the first time elucidates the mechanism of Cx26 mitochondrial translocation and its important role in the malignant progression and metastasis of CRC, which provides a new intervention target for the clinical control of highly malignant metastatic CRC and has important scientific significance for the treatment of advanced CRC. However, whether mitochondrial Cx26 localization can independently predict patient survival remains to be further validated in larger clinical cohorts with long‐term follow‐up. In addition, although our cellular and mass spectrometric evidence supports N176 as a functional site of PIMT‐mediated Cx26 modification, direct biochemical validation using purified substrates remains warranted. Future studies using synthetic peptides spanning the N176 region or soluble Cx26 fragments may provide further mechanistic validation. In addition, we discovered that BTZ had a better killing effect on highly malignant metastatic CRC, demonstrated its downstream specific molecular events, and provided a new candidate drug for the clinical treatment of highly malignant metastatic CRC, which has great application value.

## Author Contributions

**Jie Wang** & **Lu Chen**: Methodology‐most experiments; writing – original draft; formal analysis, data curation. **Xiaoning Wang**, **Sunlan Ye** & **Ning An**: Methodology‐cell experiments, data curation, Software. **Jia Qin** & **Mei Bai**: Methodology‐animal experiments, data curation. **Qiang Xu**: Conceptualization, supervision. **Xuefeng Wu** & **Yan Shen**: Supervision, writing – review & editing. All data were generated in‐house, and no paper mill was used. All authors agree to be accountable for all aspects of the work, ensuring integrity and accuracy.

## Conflicts of Interest

The authors declare no conflict of interest.

## Supporting information




**Supporting File**: advs78117‐sup‐0001‐SuppMat.docx.

## Data Availability

The data that support the findings of this study are available from the corresponding author upon reasonable request.

## References

[1] A. Calon , E. Espinet , and S. Palomo‐Ponce , “Dependency of Colorectal Cancer on a TGF‐β‐Driven Program in Stromal Cells for Metastasis Initiation,” Cancer Cell 22, no. 5 (2012): 571–584, 10.1016/j.ccr.2012.08.013. PMC3512565 23153532

[2] W. Zhang , C. Peng , and J. Yan , “Sanguisorba Officinalis L. Suppresses 5‐Fluorouracil‐Sensitive and‐Resistant Colorectal Cancer Growth and Metastasis via Inhibition of the Wnt/β‐Catenin Pathway,” Phytomedicine 94 (2022): 153844, 10.1016/j.phymed.2021.153844. 34785413

[3] M. Zeisberg and E. G. Neilson , “Biomarkers for Epithelial‐mesenchymal Transitions,” Journal of Clinical Investigation 119, no. 6 (2009): 1429–1437, 10.1172/JCI36183. PMC2689132 19487819

[4] M. Gugnoni , V. Sancisi , G. Manzotti , G. Gandolfi , and A. Ciarrocchi , “Autophagy and Epithelial–Mesenchymal Transition: An Intricate Interplay in Cancer,” Cell Death & Disease 7, no. 12 (2016): 2520, 10.1038/cddis.2016.415. PMC5260980 27929542

[5] K. Gravdal , O. J. Halvorsen , S. A. Haukaas , and L. A. Akslen , “A Switch From E‐cadherin to N‐cadherin Expression Indicates Epithelial to Mesenchymal Transition and Is of Strong and Independent Importance for the Progress of Prostate Cancer,” Clinical Cancer Research 13, no. 23 (2007): 7003–7011, 10.1158/1078-0432.CCR-07-1263. 18056176

[6] A. Kawamoto , T. Yokoe , and K. Tanaka , “Radiation Induces Epithelial‐mesenchymal Transition in Colorectal Cancer Cells,” Oncology Reports 27, no. 1 (2012): 51–57. 10.3892/or.2011.1485 21971767

[7] X. Chen , Y. Wang , and H. Xia , “Loss of E‐cadherin Promotes the Growth, Invasion and Drug Resistance of Colorectal Cancer Cells and Is Associated With Liver Metastasis,” Molecular Biology Reports 39, no. 6 (2012): 6707–6714, 10.1007/s11033-012-1494-2. 22311018

[8] L. Garcia‐Vega , E. M. O'Shaughnessy , A. Albuloushi , and P. E. Martin , “Connexins and the Epithelial Tissue Barrier: A Focus on Connexin,” Biology (Basel) 10, no. 1 (2021): 26. 10.3390/biology10010059 PMC7829877 33466954

[9] T. Aasen , S. Johnstone , L. Vidal‐Brime , K. S. Lynn , and M. Koval , “Connexins: Synthesis, Post‐Translational Modifications, and Trafficking in Health and Disease,” International Journal of Molecular Sciences 19, no. 5 (2018): 1296, 10.3390/ijms19051296. PMC5983588 29701678

[10] A. Lu , Y. Shi , and Y. Liu , “Integrative Analyses Identified Ion Channel Genes GJB2 and SCNN1B as Prognostic Biomarkers and Therapeutic Targets for Lung Adenocarcinoma,” Lung Cancer 158 (2021): 29–39, 10.1016/j.lungcan.2021.06.001. 34111567

[11] M. Delmar , D. W. Laird , C. C. Naus , M. S. Nielsen , V. K. Verselis , and T. W. White , “Connexins and Disease,” Cold Spring Harbor perspectives in biology 10, no. 9 (2018): a029348. 10.1101/cshperspect.a029348 PMC6120696 28778872

[12] M. Sun , Y. Li , and J. Qian , “Connexin26 Modulates the Radiosensitivity of Cutaneous Squamous Cell Carcinoma by Regulating the Activation of the MAPK/NF‐κB Signaling Pathway,” Frontiers in Cell and Developmental Biology 9 (2021): 672571, 10.3389/fcell.2021.672571. PMC8287175 34291047

[13] K. Ezumi , H. Yamamoto , and K. Murata , “Aberrant Expression of Connexin 26 Is Associated With Lung Metastasis of Colorectal Cancer,” Clinical Cancer Research 14, no. 3 (2008): 677–684, 10.1158/1078-0432.CCR-07-1184. 18245526

[14] H. Liu , X. Li , and C. Zhang , “GJB2 Promotes HCC Progression by Activating Glycolysis through Cytoplasmic Translocation and Generating a Suppressive Tumor Microenvironment Based on Single Cell RNA Sequencing,” Advanced Science 11, no. 39 (2024): 2402115, 10.1002/advs.202402115. PMC11497106 39162005

[15] P. S. Thiagarajan , M. Sinyuk , and S. M. Turaga , “Cx26 drives Self‐Renewal in Triple‐Negative Breast Cancer via Interaction With NANOG and Focal Adhesion Kinase,” Nature Communications 9, no. 1 (2018): 578, 10.1038/s41467-018-02938-1. PMC5805730 29422613

[16] J.‐C. Lee , S.‐U. Kang , and Y. Jeon , “Protein L‐isoaspartyl Methyltransferase Regulates p53 Activity,” Nature Communications 3, no. 1 (2012): 927, 10.1038/ncomms1933. PMC3621463 22735455

[17] J. J. Cournoyer , J. L. Pittman , and V. B. Ivleva , “Deamidation: Differentiation of Aspartyl From Isoaspartyl Products in Peptides by Electron Capture Dissociation,” Protein Science 14, no. 2 (2005): 452–463, 10.1110/ps.041062905. PMC2253403 15659375

[18] J. Zhang , Y. Li , and H. Liu , “Genome‐wide CRISPR/Cas9 Library Screen Identifies PCMT1 as a Critical Driver of Ovarian Cancer Metastasis,” Journal of Experimental & Clinical Cancer Research 41, no. 1 (2022): 24, 10.1186/s13046-022-02242-3. PMC8760697 35033172

[19] J. Wang , K. Shi , and N. An , “Direct Inhibition of GSDMD by PEITC Reduces Hepatocyte Pyroptosis and Alleviates Acute Liver Injury in Mice,” Frontiers in Immunology 13 (2022): 825428, 10.3389/fimmu.2022.825428. PMC8841757 35173734

[20] W. Guo , Y. Sun , and W. Liu , “Small Molecule‐driven Mitophagy‐mediated NLRP3 Inflammasome Inhibition Is Responsible for the Prevention of Colitis‐associated Cancer,” Autophagy 10, no. 6 (2014): 972–985, 10.4161/auto.28374. PMC4091180 24879148

[21] W. Guo , W. Liu , and Z. Chen , “Tyrosine Phosphatase SHP2 Negatively Regulates NLRP3 Inflammasome Activation via ANT1‐dependent Mitochondrial Homeostasis,” Nature Communications 8, no. 1 (2017): 2168, 10.1038/s41467-017-02351-0. PMC5735095 29255148

[22] Y. Liu , X. Ma , H. Fujioka , J. Liu , S. Chen , and X. Zhu , “DJ‐1 Regulates the Integrity and Function of ER‐mitochondria Association Through Interaction With IP3R3‐Grp75‐VDAC1,” Proceedings of the National Academy of Sciences 116, no. 50 (2019): 25322–25328, 10.1073/pnas.1906565116. PMC6911199 31767755

[23] S. Maeda , S. Nakagawa , and M. Suga , “Structure of the Connexin 26 Gap Junction Channel at 3.5 Å Resolution,” Nature 458, no. 7238 (2009): 597–602, 10.1038/nature07869. 19340074

[24] E. C. Beyer and V. M. Berthoud , “Gap Junction Structure: Unraveled, but Not Fully Revealed,” F1000Research 6 (2017): 568, 10.12688/f1000research.10490.1. PMC5414816 28529713

[25] X. Q. Gong , S. Nakagawa , T. Tsukihara , and D. Bai , “A Mechanism of Gap Junction Docking Revealed by Functional Rescue of a human‐disease‐linked Connexin Mutant,” Journal of Cell Science 126, no. Pt 14 (2013): 3113–3120. 10.1242/jcs.123430 23687377

[26] P. K. K. Mishra and M. Mahawar , “PIMT‐Mediated Protein Repair: Mechanism and Implications,” Biochemistry (Moscow) 84, no. 5 (2019): 453–463, 10.1134/S0006297919050018. 31234761

[27] T. Furuchi and H. Homma , “The Role of Isomerized Protein Repair Enzyme, PIMT, in Cellular Functions,” Yakugaku Zasshi 127, no. 12 (2007): 1927–1936, 10.1248/yakushi.127.1927. 18057781

[28] S. Yamaji , A. Droggiti , S. C. Lu , M. L. Martinez‐Chantar , A. Warner , and M. Varela‐Rey , “S‐Adenosylmethionine Regulates Connexins Sub‐types Expressed by Hepatocytes,” European Journal of Cell Biology 90, no. 4 (2011): 312–322, 10.1016/j.ejcb.2010.09.007. PMC3042521 21093098

[29] D. W. Horne , R. S. Holloway , and C. Wagner , “Transport of S‐adenosylmethionine in Isolated Rat Liver Mitochondria,” Archives of Biochemistry and Biophysics 343, no. 2 (1997): 201–206, 10.1006/abbi.1997.0167. 9224731

[30] G. Agrimi , M. A. Di Noia , C. M. Marobbio , G. Fiermonte , F. M. Lasorsa , and F. Palmieri , “Identification of the human Mitochondrial S‐adenosylmethionine Transporter: Bacterial Expression, Reconstitution, Functional Characterization and Tissue Distribution,” Biochemical Journal 379, no. 1 (2004): 183–190, 10.1042/bj20031664. PMC1224042 14674884

[31] S. K. Joseph , D. M. Booth , M. P. Young , and G. Hajnoczky , “Redox Regulation of ER and Mitochondrial Ca^2+^ Signaling in Cell Survival and Death,” Cell Calcium 79 (2019): 89–97, 10.1016/j.ceca.2019.02.006. PMC8409742 30889512

[32] E. Bertero and C. Maack , “Calcium Signaling and Reactive Oxygen Species in Mitochondria,” Circulation Research 122, no. 10 (2018): 1460–1478, 10.1161/CIRCRESAHA.118.310082. 29748369

