# Supplementary material for: Mitochondrial Localization of Cx26 Promotes Colorectal Cancer Malignancy and Exposes a Fatal Vulnerability to Drug Targeting
Source: Adv Sci (Weinh). 2026 Sep 30:e78117. Online ahead of print. doi: 10.1002/advs.78117 (PMC13626670; doi:10.1002/advs.78117)
Supplement: Supplementary file 1 [file ADVS-9999-e78117-s001.docx]

**Supplementary figure legends**

**Supplementary figure 1.** **Cx26 is associated with the survival probability in patients of colorectal cancer.** (A-B) The relationship between Cx26 expression with survival probability in two GEO databases, respectively (GEO database ID: 17536, 17538).

**Supplementary figure 2. SW620 cells exhibit higher degree of malignancy than SW480 cells.** (A) SW480 and SW620 cells (5×10^4^ cells/well) were seeded in 96-well plates, cell growth was examined by MTT assay for indicated times. ^*^*P* < 0.05 *vs*. SW480; by two-way ANOVA (mean ± SEM). (B) Colony formation assay was performed after SW480 and SW620 cells were cultured for 14 days. (C) Colony number was calculated with Image J software. ^**^*P* < 0.01 *vs*. SW480; by unpaired student’s *t*-test (mean ± SEM). (D) Representative images of morphological characteristics of SW480 and SW620 cells. (E) The expression of E-cadherin, Vimentin and N-cadherin was detected by western blot and Tubulin was tested as a loading control. (F) The expression of CD133 in SW480 and SW620 cells was examined by FACS. (G) The mRNA level of *Lgr5* in SW480 and SW620 cells was examined by real-time PCR. ^*^*P* < 0.05 *vs*. SW480; by unpaired student’s *t*-test (mean ± SEM).

**Supplementary figure 3.** **Subcellular localization of Cx26.** (A) NCM460, SW480 and SW620 cells were stained with anti-Cx26 antibody, and then analyzed by FACS. (B) Western blot of Cx26, Calreticulin, GM130 and EEA1 protein levels in ER-, golgi- and endosome-enriched fractions of SW480 and SW620 cells. Calreticulin, GM130 and EEA1 were measured as loading controls of ER-, golgi- and endosome-enriched fractions respectively. (C) Immunoblot of HA, TOM20, Hsp60 and GAPDH in the cytoplasm and submitochondrial fractions of HEK293T cells transfected with or without Cx26-HA plasmid. TOM20, Hsp60 and GAPDH were measured as loading controls of mitochondrial outer membrane (MM), mitochondrial matrix and cytoplasm respectively. (D) Western blot of E-cadherin, N-cadherin, Vimentin, Cx26 and Tubulin protein levels in B16F1 and B16F10 cells. Tubulin was measured as loading control. (E) Immunoblot of Cx26, Na,K-ATPase and Tubulin in cytoplasm and membrane of B16F1 and B16F10 cells. Na,K-ATPase and Tubulin were measured as loading control of membrane and cytoplasm respectively. (F) Western blot of Cx26, COX4, Tubulin and Calnexin protein levels in cytoplasmic and mitochondria-enriched fractions of B16F1 and B16F10 cells. Tubulin and COX4 were used as markers and loading controls for the cytoplasmic and mitochondria-enriched fractions, respectively. Results shown are representative of three replicates.

**Supplementary figure 4.** **The localization of Cx26 is not related to its N-terminal 40 amino acids.** (A) The signal peptide of Cx26 was predicted by SignalP-5.0. (B) The expression levels of HA, Tubulin and Na,K-ATPase in the cytoplasm and membrane of HEK293T transfected with Cx26-HA or Cx26 Δ1-40-HA plasmid. Na,K-ATPase and Tubulin were measured as loading controls of membrane and cytoplasm respectively. Data shown are representative of three replicates.

**Supplementary figure 5.** **PIMT is associated with the malignant progression in patients of colorectal cancer.** (A-B) The relationship between malignant progressions of colorectal cancer with PIMT expression was analyzed. (C) The association of PIMT expression in human colorectal cancer was found with the overall survival probability.

**Supplementary figure 6.** **PIMT expression is positively associated with the malignant progression of CRC.** (A-B) Representative immunohistochemistry images and histology scores of PIMT in clinical CRC tissue microarray. Scale bar, 100 μm. ^*^*P* < 0.05, ^**^*P* < 0.01 *vs*. Normal; by Welch’s one-way ANOVA followed by Dunnett’s T3 multiple comparisons test. (mean ± SEM).

**Supplementary figure 7. PIMT interacts with Cx26 and suppresses gap junction intercellular communication in highly metastatic tumor cells.** (A) Lysates of B16F1 and B16F10 cells were co-immunoprecipitation with anti-PIMT antibody, and then PIMT and Cx26 protein levels were detected by immunoblot. (B-C) The GJIC function in SW620-scramble and SW620-shRNA-PIMT cells was tested, and the number of receiver cells around each donor cell was calculated. ^**^*P* < 0.01 *vs*. SW620-Scramble; by unpaired student’s *t*-test (mean ± SEM).

**Supplementary figure 8. Cx26 in SW620 cells is more stable than in SW480 cells.** (A-C) The protein levels of Cx26 and PIMT were analyzed by western blot and statistical analysis in SW480 and SW620 cells treated with CHX (100 μM) for 0, 3, 6, 12, 24 h. GAPDH and Tubulin were used as loading controls respectively. ^*^*P* < 0.05, ^**^*P* < 0.01 *vs*. SW480; by two-way ANOVA followed by Šídák’s multiple comparisons test (mean ± SEM).

**Supplementary figure 9. Cx26 can improve mitochondrial Ca^2+^ uptake and EMT.** (A) Representative curves and statistical analysis of mitochondrial Ca^2+^ before and after histamine (20 μM) treatment in HEK293T-pCMV-CEPIA4-mit cells transfected with or without Cx26-HA plasmid. ^***^*P* < 0.001 *vs*. NC; by unpaired student’s *t*-test (mean ± SEM). (B) The expression levels of E-cadherin, N-cadherin, Vimentin, Flag and Tubulin in SW480 cells transfected with Cx26-Flag (0, 1, 2 μg) were analyzed by western blot and statistical analysis, and Tubulin was used as a loading control. ^**^*P* < 0.01, ^***^*P* < 0.001 *vs*. 0 μg; by one-way ANOVA followed by Dunnett’s multiple comparisons test (mean ± SEM). (C) The expression levels of E-cadherin, N-cadherin, Vimentin, Cx26 and Tubulin in SW620 cells transfected with shRNA-NC or shRNA-Cx26 were analyzed by western blot and statistical analysis, and Tubulin was used as a loading control. ^*^*P* < 0.05, ^**^*P* < 0.01, ^***^*P* < 0.001 *vs*. shRNA-NC; by unpaired student’s *t*-test (mean ± SEM).

**Supplementary figure 10.** **Overexpression of Cx26 promotes the proliferation and metastasis of SW480 cells.** (A) The expression levels of Cx26 and Tubulin in SW480-NC and SW480-OE-Cx26 cells were analyzed by western blot, and Tubulin was used as a loading control. ^**^*P* < 0.01 *vs*. NC; by unpaired student’s *t*-test (mean ± SEM). (B) Representative images and volume growth curves of tumors established by SW480-NC and SW480-OE-Cx26 cells (*n* = 6). ^*^*P* < 0.05 *vs*. NC; by two-way repeated-measures ANOVA with Geisser-Greenhouse correction (mean ± SEM). (C) Weight of tumors established by SW480-NC and SW480-OE-Cx26 cells (*n* = 6). ^*^*P* < 0.05 *vs*. NC; by unpaired student’s *t*-test (mean ± SEM). (D) The expression levels of N-cadherin, E-cadherin, Vimentin, Cx26 and Tubulin in tumors established by SW480-NC and SW480-OE-Cx26 cells were analyzed by western blot and statistical analysis, and Tubulin was used as a loading control. ^**^*P* < 0.01, ^***^*P* < 0.001 *vs*. NC; by unpaired student’s *t*-test (mean ± SEM). (E) Representative H&E-stained sections of lung tissues from mice intravenously injected with SW480 cells expressing NC or OE-Cx26. Metastatic area was quantified using QuPath (*n* = 6). ^**^*P* < 0.01 *vs*. NC; by unpaired student’s *t*-test (mean ± SEM).

**Supplementary figure 11.** **Cx26 knockdown inhibits the proliferation and metastasis of B16F10 cells.** (A-B) The expression levels of Cx26 and Tubulin in B16F10-shRNA-NC and B16F10-shRNA-Cx26 cells were analyzed by western blot, and Tubulin was used as a loading control. ^***^*P* < 0.001 *vs*. shRNA-NC; by unpaired student’s *t*-test (mean ± SEM). (C) Volume growth curves of tumors established by B16F10-shRNA-NC and B16F10-shRNA-Cx26 cells (*n* = 5). ^*^*P* < 0.05 *vs*. shRNA-NC; by two-way repeated-measures ANOVA with Geisser-Greenhouse correction (mean ± SEM). (D) Weight of tumors established by B16F10-shRNA-NC and B16F10-shRNA-Cx26 cells (*n* = 5). ^*^*P* < 0.05 *vs*. shRNA-NC; by unpaired student’s *t*-test (mean ± SEM). (E) The expression levels of N-cadherin, E-cadherin, Vimentin, Cx26 and Tubulin in tumors established by B16F10-shRNA-NC and B16F10-shRNA-Cx26 cells were analyzed by western blot and statistical analysis, and Tubulin was used as a loading control. ^***^*P* < 0.001 *vs*. shRNA-NC; by unpaired student’s *t*-test (mean ± SEM). (F) Representative gross images and H&E-stained sections of lung tissues from mice intravenously injected with B16F10 cells expressing shRNA-NC or shRNA-Cx26 are shown. The number of macroscopic metastatic nodules per lung was quantified (*n* = 5). T, tumor; NL, normal lung tissue. Scale bar, 500 μm. ^*^*P* < 0.05 *vs*. shRNA-NC; by unpaired student’s *t*-test (mean ± SEM).

**Supplementary figure 12.** **Both RR and MCU-i4 downregulate EMT signaling in CRC cells.** (A) Immunoblot of E-cadherin, N-cadherin, Vimentin, and Tubulin in SW620 cells treated with or without RR (10 μM) for 24 h. Tubulin were performed as loading controls, respectively. Relative E-cadherin, N-cadherin and Vimentin levels were normalized to Tubulin. ^**^*P* < 0.01, ^***^*P* < 0.001 *vs*. DMSO; by unpaired student’s *t*-test (mean ± SEM). (B) Immunoblot of E-cadherin, N-cadherin, Vimentin, and Tubulin in SW620 cells treated with or without MCU-i4 (10 μM) for 24 h. (C)The mRNA levels of *GJB2* were detected in SW480 cells transfected with Cx26 WT-HA or Cx26 N176A-HA plasmid. Data were normalized to *GAPDH*. ^***^*P* < 0.001 *vs*. Control; by one-way ANOVA followed by Dunnett’s multiple comparisons test (mean ± SEM).

**Supplementary figure 13.** **SW620 cells are more sensitive to BTZ than SW480 cells.** (A-F) The IC_50_ values of 5-Fu, oxaliplatin, irinotecan, paclitaxel, etoposide and BTZ in SW480 and SW620 cells were measured by MTT assay. Data are presented as mean ± SEM of three replicates.

**Supplementary figure 14.** **B16F10 cells are more sensitive to BTZ than B16F1 cells.** (A) The IC_50_ values of BTZ in B16F1 and B16F10 cells were measured by MTT assay. Data are presented as mean ± SEM from at least three independent experiments.

**Supplementary figure 15.** **The proliferation and migration of SW620 cells are more significantly inhibited than SW480 cells by BTZ.** (A) The IC_50_ values of BTZ in SW480 and SW620 cells were measured by MTT assay. (B) Representative images of SW480 and SW620 cells treated with BTZ (0, 10, 30, 100 nM) for 24 h. Scale bar, 200 μm. (C) Representative images and statistical analysis of colony formation for SW480 and SW620 cells treated with BTZ (0, 10, 30, 100 nM). ns means no significant difference, ^**^*P* < 0.01, ^***^*P* < 0.001 *vs*. SW480 (BTZ 0 nM), ^##^*P* < 0.01, ^###^*P* < 0.001 *vs*. SW620 (BTZ 0 nM); by two-way ANOVA followed by Dunnett’s multiple comparisons test (mean ± SEM).

 **Supplementary figure 16.** **BTZ suppresses EMT, invasion, and metastasis in CRC cells.** (A-C) Representative Western blot images and quantitative analysis of N-cadherin and Vimentin expression in SW480 and SW620 cells treated with BTZ (0, 10, 100 nM). Actin was used as the loading control. ns means no significant difference, ^*^*P* < 0.05, ^**^*P* < 0.01 *vs*. SW480 (BTZ 0 nM), ^#^*P* < 0.05, ^###^*P* < 0.001 *vs*. SW620 (BTZ 0 nM); by two‑way ANOVA followed by Dunnett’s multiple comparisons test (mean ± SEM). (D) Representative images and quantitative analysis of Transwell invasion assays in SW480-NC and SW480-OE-Cx26 cells treated with BTZ (0, 10, 100 nM). Scale bar, 50 μm. ns means no significant difference, ^**^*P* < 0.01 *vs*. SW480-NC (BTZ 0 nM), ^##^*P* < 0.01, ^###^*P* < 0.001 *vs*. SW480-OE-Cx26 (BTZ 0 nM); by two‑way ANOVA followed by Dunnett’s multiple comparisons test (mean ± SEM). (E) Representative H&E-stained sections of lung tissues from mice injected with SW620 cells via the tail vein with or without BTZ treatment (1 mg/kg). Metastatic area was quantified using QuPath (*n* = 6). ^**^*P* < 0.01 *vs*. NC; by unpaired student’s *t*-test (mean ± SEM).

**Supplementary figure 17.** **Differences in BTZ sensitivity in SW480 and SW620 cells are independent of their proteasome activity.** (A) Relative proteasome activities of SW480 and SW620 cells were detected by proteasome activity assay kit. ns means no significant difference; by unpaired student’s *t*-test (mean ± SEM). (B) Relative proteasome activities of SW480 and SW620 cells treated with BTZ (0, 10, 100 nM) were detected by proteasome activity assay kit. ns means no significant difference, ^*^*P* < 0.05 *vs*. SW480 (BTZ 10 nM); by two-way ANOVA followed by Šídák’s multiple comparisons test (mean ± SEM). (C) The IC_50_ values of MG132 in SW480 and SW620 cells were measured by MTT assay.

**Supplementary figure 18. BTZ can interact with PIMT.** (A-B) SW620 cells were incubated with DMSO or BTZ (100 nM) for 2 h. Cells were harvested and divided into 10 aliquots and heated at indicated temperatures. The supernatant of each temperature was then analyzed by western blot. (C) Molecular docking analysis of BTZ and 20S proteasome via Discovery studio. On the right is the 2D diagram of the interaction between BTZ and amino acid residues of 20S proteasome. (D-E) SW620 cells were incubated with DMSO or BTZ (100 nM) for 2 h. Cells were harvested and divided into 10 aliquots and heated at indicated temperatures. The supernatant of each temperature was then analyzed by western blot. Illustration of the remaining amount of PIMT was examined. ^**^*P* < 0.01, ^***^*P* < 0.001 *vs*. DMSO; by two-way ANOVA followed by Šídák’s multiple comparisons test (mean ± SEM).

**Supplementary figure 19.** **The level of intracellular Ca^2+^ increased in SW620 cells is more than that in SW480 cells after BTZ treatment.** (A-B) Flow cytometry analysis of intracellular Ca^2+^ by Fluo-4 AM staining in SW480 and SW620 cells treated with BTZ (0, 10, 30, 100 nM) for 24 h. ^**^*P* < 0.01 *vs*. SW480 (BTZ 0 nM), ^###^*P* < 0.001 *vs*. SW620 (BTZ 0 nM); by two‑way ANOVA followed by Dunnett’s multiple comparisons test (mean ± SEM).

**Supplementary figure 20.** **Cx26 negatively regulates MAPK signaling pathway.** (A) The GSEA analysis of MAPK signaling pathway.

**Supplementary figure 21.** **BTZ significantly increases the expression level of p-ERK in SW620 cells.** (A-D) Immunoblot and statistical analysis of p-ERK, p-p38, p-Jnk and Tubulin in SW480 and SW620 cells treated with BTZ (0, 10, 30, 100 nM) for 24 h. Tubulin was used as a loading control. ns means no significant difference, ^***^*P* < 0.001 *vs*. SW480 (BTZ 0 nM), ^#^*P* < 0.05, ^###^*P* < 0.001 *vs*. SW620 (BTZ 0 nM); by two‑way ANOVA followed by Dunnett’s multiple comparisons test (mean ± SEM).

**Supplementary figure 22. BTZ is more likely to induce apoptosis and autophagy in SW620 cells than SW480 cells.** (A) SW480 and SW620 cells were treated with BTZ (0, 10, 100 nM) for 24 h, and then cells stained with Annexin V and PI were detected by flow cytometry. (B-C) Immunoblot of PARP, cleaved PARP, LC3-I, LC3-II and Tubulin in SW480 and SW620 cells incubated with BTZ (0, 10, 30, 100 nM) for 24 h. PARP and Tubulin were used as a loading control, respectively. Relative cleaved PARP levels were normalized to PARP. Relative LC3-II levels were normalized to Tubulin. ns means no significant difference, ^**^*P* < 0.01, ^***^*P* < 0.001 *vs*. SW480 (BTZ 0 nM), ^##^*P* < 0.01, ^###^*P* < 0.001 *vs*. SW620 (BTZ 0 nM); by two‑way ANOVA followed by Dunnett’s multiple comparisons test (mean ± SEM). (D) The mRNA levels of *BCL2L1*, *MDR*1 and *p62* were detected in SW480 and SW620 cells treated with BTZ (0, 10, 30, 100 nM) for 24 h. Results were normalized to *GAPDH*. ns means no significant difference, ^**^*P* < 0.01, ^***^*P* < 0.001 *vs*. SW480 (BTZ 0 nM), ^#^*P* < 0.05, ^###^*P* < 0.001 *vs*. SW620 (BTZ 0 nM); by two‑way ANOVA followed by Dunnett’s multiple comparisons test (mean ± SEM).

**Supplementary figure 23.** **PD0325901 significantly reduces BTZ-induced apoptosis and autophagy in SW620 cells.** (A-B) The IC_50_ values of BTZ in SW480 and SW620 cells treated with or without PD0325901 (10 μM) were measured by MTT assay. Data are presented as mean ± SEM of three replicates. (C-F) SW620 cells were treated with BTZ (0, 10, 30, 100 nM) alone or combined with PD0325901 (10 μM) for 24 h. The protein levels of p-ERK, PARP, cleaved PARP, BCL2, LC3-I, LC3-II and Tubulin were measured by western blot, and Tubulin was performed as a loading control. The mRNA levels of *BCL2L1*, *MDR1*, *p62* were detected by QPCR, and data were normalized to *GAPDH*. ns means no significant difference, ^**^*P* < 0.01, ^***^*P* < 0.001 *vs*. SW620 (BTZ 0 nM), ^##^*P* < 0.01, ^###^*P* < 0.001 *vs*. SW620 (BTZ 0 nM)+PD0325901; by two‑way ANOVA followed by Dunnett’s multiple comparisons test (mean ± SEM). (G-J) SW480 cells were treated with BTZ (0, 10, 30, 100 nM) alone or combined with PD0325901 (10 μM) for 24 h. The protein levels of p-ERK, PARP, cleaved PARP, BCL2, LC3-I, LC3-II and Tubulin were measured by western blot, and Tubulin was performed as a loading control. The mRNA levels of *BCL2L1*, *MDR1*, *p62* were detected by QPCR, and data were normalized to *GAPDH*. ns means no significant difference, ^*^*P* < 0.05, ^**^*P* < 0.01, ^***^*P* < 0.001 *vs*. SW480 (BTZ 0 nM), ^#^*P* < 0.05, ^##^*P* < 0.01, ^###^*P* < 0.001 *vs*. SW480 (BTZ 0 nM)+PD0325901; by two‑way ANOVA followed by Dunnett’s multiple comparisons test (mean ± SEM).

**Supplementary figure 24.** **Excessive mitochondrial Ca^2+^ uptake stimulates ROS production.** (A-B) Flow cytometry analysis of intracellular ROS by DCFH-DA staining in SW480 and SW620 cells treated with RR (10 μM), BTZ (100 nM) or RR+BTZ. (C-D) Flow cytometry analysis of intracellular Ca^2+^ by Fluo-4 AM staining in SW480 and SW620 cells treated with NAC (10 mM), BTZ (100 nM) or NAC+BTZ. ns means no significant difference, ^*^*P* < 0.05, ^***^*P* < 0.001 *vs*. SW480 or SW620, ^#^*P* < 0.05, ^##^*P* < 0.01, ^###^*P* < 0.001 *vs*. SW480+BTZ or SW620+BTZ; by two-way ANOVA followed by Šídák’s multiple comparisons test (mean ± SEM).

**Supplementary figure 25. NAC significantly inhibits BTZ-induced apoptosis in SW620 cells.** (A-B) Flow cytometry analysis of cell apoptosis by Annexin-V and PI staining in SW480 and SW620 cells treated with NAC (10 mM), BTZ (100 nM) or NAC+BTZ for 24 h. ^**^*P* < 0.01, ^***^*P* < 0.001 *vs*. SW480 or SW620, ^##^*P* < 0.01, ^###^*P* < 0.001 *vs*. SW480+BTZ or SW620+BTZ; by two-way ANOVA followed by Šídák’s multiple comparisons test (mean ± SEM).
